# Functional characterization of probiotic surface layer protein-carrying *Lactobacillus amylovorus* strains

**DOI:** 10.1186/1471-2180-14-199

**Published:** 2014-07-28

**Authors:** Ulla Hynönen, Ravi Kant, Tanja Lähteinen, Taija E Pietilä, Jasna Beganović, Hauke Smidt, Ksenija Uroić, Silja Åvall-Jääskeläinen, Airi Palva

**Affiliations:** 1 Department of Veterinary Biosciences, Division of Microbiology and Epidemiology, University of Helsinki, P.O. Box 66, Helsinki 00014, Finland; 2 Faculty of Food Technology and Biotechnology, Laboratory of Antibiotic, Enzyme, Probiotic and Starter Culture Technologies, University of Zagreb, Pierottijeva 6, Zagreb 10000, Croatia; 3 Laboratory of Microbiology, Wageningen University, Dreijenplein 10, Wageningen NL-6703 HB, The Netherlands

**Keywords:** S-layer, *Lactobacillus*, Adhesion, IPEC-1, Dendritic cell

## Abstract

**Background:**

Adhesiveness to intestinal epithelium, beneficial immunomodulating effects and the production of pathogen-inhibitory compounds are generally considered as beneficial characteristics of probiotic organisms. We showed the potential health-promoting properties and the mechanisms of probiotic action of seven swine intestinal *Lactobacillus amylovorus* isolates plus the type strain (DSM 20531^T^) by investigating their adherence to porcine intestinal epithelial cells (IPEC-1) and mucus as well as the capacities of the strains to i) inhibit the adherence of *Escherichia coli* to IPEC-1 cells, ii) to produce soluble inhibitors against intestinal pathogens and iii) to induce immune signaling in dendritic cells (DCs). Moreover, the role of the *L. amylovorus* surface (S) –layers - symmetric, porous arrays of identical protein subunits present as the outermost layer of the cell envelope - in adherence to IPEC-1 cells was assessed using a novel approach which utilized purified cell wall fragments of the strains as carriers for the recombinantly produced S-layer proteins.

**Results:**

Three of the *L. amylovorus* strains studied adhered to IPEC-1 cells, while four strains inhibited the adherence of *E. coli*, indicating additional mechanisms other than competition for binding sites being involved in the inhibition. None of the strains bound to porcine mucus. The culture supernatants of all of the strains exerted inhibitory effects on the growth of *E. coli*, *Salmonella*, *Listeria* and *Yersinia*, and a variable, strain-dependent induction was observed of both pro- and anti-inflammatory cytokines in human DCs. *L. amylovorus* DSM 16698 was shown to carry two S-layer-like proteins on its surface in addition to the major S-layer protein SlpA. In contrast to expectations, none of the major S-layer proteins of the IPEC-1 -adhering strains mediated bacterial adherence.

**Conclusions:**

We demonstrated adhesive and significant pathogen inhibitory efficacies among the swine intestinal *L. amylovorus* strains studied, pointing to their potential use as probiotic feed supplements, but no independent role could be demonstrated for the major S-layer proteins in adherence to epithelial cells. The results indicate that many intestinal bacteria may coexist with and confer benefits to the host by mechanisms not attributable to adhesion to epithelial cells or mucus.

## Background

Porcine diarrhea during the neonatal and weaning periods is a common problem in pig production farms, resulting in significant mortality and economic losses for pig farmers. The aetiology of post-weaning diarrhea is multifactorial, but an elevated susceptibility to enteric infections due to the altered intestinal microbial balance is believed to play a central role [1]. The micro-organisms most often implicated in post-weaning diarrhea are enterotoxigenic *E. coli* (ETEC) strains expressing F4 (K88), F5, F6 or F18 fimbriae [2,3]. In the management of piglet gut health around weaning, feed supplementation with lactobacilli has proved beneficial [4-7], although in many cases the molecular mechanisms underpinning the beneficial effects have remained unknown. As adhesion to host tissues is essential for many gastro-intestinal pathogens, the paradigm of competitive exclusion through competition for binding sites has evolved. Therefore, knowledge about *Lactobacillus* surface components and their roles as adhesins is of major importance when developing strategies based on the administration of commensal bacteria to promote piglet health.

S (surface) layers are the outermost cell envelope structures commonly found on the surface of lactobacilli and other bacterial species. They are composed of numerous identical (glyco)protein subunits (with a 25–71 kDa size in lactobacilli), which form a regular, symmetric and porous array, completely covering the bacterial cell surface. The subunits are held together and connected to the underlying cell surface by non-covalent interactions, and they spontaneously reassemble *in vitro* by an entropy-driven process, i.e. the subunit proteins are very poorly water-soluble [8]. The biological functions of *Lactobacillus* S-layer proteins (Slp:s) are not well understood. In some *Lactobacillus* species, as well as in many other bacteria, S-layer proteins mediate bacterial adherence to host cells or to the extracellular matrix [9-19], but in most cases, the functions of *Lactobacillus* S-layer proteins have remained unknown.

Unlike in humans, lactobacilli are an essential component of the gastrointestinal microbiota of swine [20,21], with *L. amylovorus* representing a characteristic species which is especially abundant in piglets [22,23]. The S-layer carrying *L. amylovorus* strain DSM 16698, isolated from the small intestine of a piglet [24,25], has been shown to exhibit potentially health-promoting effects both *in vitro* and in weaned piglets *in vivo*[26,27]. This has raised the question if other strains of this commensal species might also have the potential to be used as probiotic feed additives during the weaning period. Furthermore, another question is related to the role of Slp:s in the probiotic effects of *L. amylovorus*. We have previously isolated several surface-layer carrying *L. amylovorus* strains from the small intestine or faeces of pigs and preliminarily characterized them for their putative probiotic properties [28,29]. While simultaneously carrying out the whole genome sequencing of the strains (Kant et al., manuscript in preparation), the present study was undertaken to characterize in detail the putative probiotic properties of these strains and to reveal the role of their divergent S-layer proteins in adherence to porcine intestinal epithelium *in vitro*. As a comparison, the above-mentioned *L. amylovorus* strain DSM 16698 of swine intestinal origin, and DSM 20531^T^, an S-layer carrying strain isolated from silage, were included in the experiments.

## Methods

### Bacterial strains and culture conditions

The bacterial strains used in this study are listed in Table 1. *Lactobacillus* strains were cultivated anaerobically in MRS-broth (Difco, BD, Franklin Lakes, New Jersey) at +37°C. Enterotoxigenic *Escherichia coli* (ETEC) was cultured in Luria-Bertani (LB) broth (Difco, BD) with agitation at +37°C in the experiments assessing the effect of *Lactobacillus* strains on ETEC adherence. The *E. coli* strains used for the cloning and expression of Slp-encoding genes were cultivated with agitation in LB broth, or in the heterologous gene expression, in M9ZB medium [30] at +37°C, with kanamycin (30 μg/ml) being added when appropriate. In the pathogen inhibition assays, all the pathogens were cultivated in tryptic soy agar (TSA) plates (Difco) and subcultured in tryptic soy broth (TSB, Difco) at +37°C with agitation.

**Table 1:** Strains used in this study

**Strain**	**Reference or source**	**S-layer protein**	
		**Name**	**Calculated Mw**^a^**(kDa)**
*Lactobacillus amylovorus* DSM 16698	porcine faeces [24]	SlpA	44
		SlpB	50
		SlpC	40
*Lactobacillus amylovorus* DSM 20531^T^	DSM, fermented corn silage	SlpA	44
*Lactobacillus amylovorus* GRL 1112 (LAB 2)	porcine faeces [28]	SlpA	45
*Lactobacillus amylovorus* GRL 1114 (LAB 8)	porcine faeces [28]	SlpA	43
*Lactobacillus amylovorus* GRL 1115 (LAB 13)	porcine ileum [28]	SlpA	47
*Lactobacillus amylovorus* GRL 1116 (LAB 16)	porcine jejunum [28]	SlpA	46
*Lactobacillus amylovorus* GRL 1117 (LAB 31)	porcine jejunum [28]	SlpA	61
		SlpB^b^	49
*Lactobacillus amylovorus* GRL 1118 (LAB 52)	porcine jejunum [28]	SlpA	48
*Escherichia coli* F4^+^ (ETEC)	[26]	-^c^	-^c^
*Escherichia coli* ERF 2014; O141, F18^+^	DVB^d^	-^c^	-^c^
*Escherichia coli* ATCC 43894; O157 (EHEC)	ATCC	-^c^	-^c^
*Salmonella typhimurium* ATCC14028	ATCC	-^c^	-^c^
*Listeria monocytogenes* R14-2-2	DVB^d^	-^c^	-^c^
*Yersinia enterocolitica* R5-9-1	DVB^d^	-^c^	-^c^
*Escherichia coli* DH5αF’	[31]	-^c^	-^c^
*Escherichia coli* BL21 (DE3)	EMD Millipore	-^c^	-^c^

^a^molecular weight of mature protein ^b^not tested for adherence ^c^not present ^d^culture collection of the Department of Veterinary Biosciences/Veterinary Microbiology and Epidemiology, University of Helsinki, Finland.

### Cell culture

The non-transformed continuous cell line IPEC-1, isolated from the small intestine of an unsuckled, newborn piglet [32] was used as a model for porcine small intestinal epithelium. The cells were cultured in Dulbecco’s modified eagle medium/Ham’s F12 Nutrient Mixture (DMEM/Ham’s F-12 [1:1]) supplemented with 5% fetal calf serum (FCS), 1% insulin-transferrin-selenium (ITS), 16 mmol/L HEPES (all PAN-Biotech, Germany) and 5 ng/mL epidermal growth factor (EGF; BD, Franklin Lakes, New Jersey) at 39°C and 5% CO_2_. In the adhesion and adhesion inhibition experiments, the cells were seeded at a density of 2 x 10^5^ /ml to a Transwell-like culture (Thincerts™, 1 μm pore size, diameter 10 mm; Greiner bio-one, Frickenhausen, Germany) and cultured for 4–5 days to allow differentiation, until the transepithelial electric resistance (TEER) value was ≥1 kΩcm^2^.

### Detection and expression analysis of *slp* genes in *L. amylovorus*

The presence of S-layer proteins on the surface of the *L. amylovorus* intestinal isolates GRL 1112 – GRL 1118 has previously been described [28]. The putative *slp* encoding genes were identified *in silico* in the draft genomes of the *L. amylovorus* strains based on homology with the publicly available *L. acidophilus slp* gene sequences. The identification of the expressed *slp* genes was based on the observed molecular weights of the proteins, obtained by analyzing overnight cultures of the strains by standard SDS-PAGE in 12% gels, and on the amino-terminal and/or internal amino acid sequences of the Slp:s. The amino-terminal sequences were obtained by an Edman-degradation-based Procise 494 HT sequencer (Life Technologies, Carlsbad, CA), and internal peptide sequences through a peptide mapping analysis: the proteins were digested in-gel by trypsin followed by analysis with liquid chromatography coupled to tandem mass spectrometry (LC-MS/MS) carried out with an EASY-nLC liquid chromatograph (Thermo Fisher Scientific, Germany) connected to a Velos Pro-Orbitrap Elite hybrid mass spectrometer (Thermo Fisher Scientific, Germany) with a nano-electrospray ion source (Thermo Fisher Scientific, Germany). Both amino-terminal sequencing and peptide mapping were performed in the Institute of Biotechnology (University of Helsinki, Finland).

### Cloning and heterologous expression of the genes encoding *L. amylovorus* S-layer proteins

The expressed *slp* genes (see above) were amplified by PCR from the chromosomal DNA of the *L. amylovorus* strains, cloned as *Nco*I-*Xho*I –fragments in *E. coli* DH5αF', sequenced to verify the correct open reading frames, and expressed in *E. coli* BL21 (DE3) as C-terminal hexahistidine tag-fusions, as described in the pET system manual (Merck KGaA, Darmstadt, Germany) and as previously reported [33]. Recombinant S-layer proteins were purified in the presence of 4 M guanidine hydrochloride (GuHCl) with His Trap HP columns (GE Healthcare, Little Chalfont, UK) according to the manufacturer’s instructions. The pooled protein fractions were dialysed against deionized water overnight at +4°C, centrifuged (15 000 g, 20 min, +4°C) and stored in aliquots at -80°C.

### Purification of porcine intestinal mucus

The 8-week old pig used for mucus isolation was housed in a piggery of MTT Agrifood Research (Finland), treated in strict accordance with the recommendations of the Finnish Ministry of Agriculture and Forestry (Directive 2013–497) and EEC (Directive 86/609/EEC) for the care and use of animals in research, and sacrificed by bolt gun. As the pig used in this study was not specifically included in any experimental protocol on living animals before slaughtering, there was no ethical requirement for collecting mucus samples. The mucus isolation protocol was modified from [34]. Briefly, the small intestine was opened longitudinally and washed with cold phosphate-buffered saline (PBS) with 0.1 mM phenylmethylsulfonyl fluoride (PMSF) as a protease inhibitor (PBS-PMSF). Mucus was collected by gentle scraping into PBS-PMSF, centrifuged (17,000 g, 1 hour, +4°C) to remove cells and insoluble material, and the supernatant was homogenized in a domestic blender. The homogenate was concentrated in a Centricon Plus-70 filter unit (molecular weight cutoff 10,000), clarified by centrifugation (17,000 g, 30 min, +4°C), filtered twice through a glass fibre filter (GE Healthcare, Little Chalfont, UK) and once through a 0.8 μm cellulose acetate filter (Sartorius, Goettingen, Germany) and purified by gel filtration chromatography at +4°C in a Sephacryl S-200 HiPrep 16/60 column (GE Healthcare, Little Chalfont, UK) at a flow rate of 1.8 ml/min with PBS as the eluent, monitoring the A_280nm_ values of 5 ml fractions. The protein-containing fractions were dialyzed against water and assayed for total protein by the method of Bradford (Bio-Rad Protein Assay, Bio-Rad, Hercules, CA) using bovine serum albumin (BSA) as a standard, and for glycoproteins using the Crypton™ Glycoprotein Staining Kit (Thermo Scientific, Waltham, MA), with porcine gastric mucins (Sigma), horseradish peroxidase (HRP) and soybean trypsin inhibitor (Thermo Scientific, Waltham, MA) as standards. The void volume fractions with a high glycoprotein content were pooled, lyophilized and stored at -20°C.

### Adherence of *L. amylovorus* strains to mucus

The adherence of the *L. amylovorus* strains to porcine gastric mucins (type II, Sigma) or to porcine small intestinal mucus was studied essentially as described earlier [35], but by using a nucleic acid binding fluorescent stain SYTO^®^9 (Molecular Probes, Eugene, OR) rather than tritium for bacterial labeling, and PBS as the buffer. To label the bacterial cells in the experiments, the strains were cultivated overnight, collected, washed twice with 0.85% NaCl and suspended into the original volume of 0.85% NaCl, and then 1 μl of 5 mM SYTO^®^9 solution was added per 1 ml of cell suspension, followed by a 15 minute incubation in the dark with vigorous shaking, after which the cells were collected and washed twice with PBS. After the adherence assay, the input (added) and output (remaining) fluorescence values were measured in a microplate reader (Victor Multilabel Plate Reader, Perkin Elmer, Waltham, MA) and the adherence was expressed as the proportion (%) of the original fluorescence remaining, after first subtracting the background fluorescence from mucus-coated wells without bacteria (for outputs) and from wells filled with PBS (for inputs).

### Adherence of *L. amylovorus* strains to IPEC-1 cells

*L. amylovorus* strains were cultivated overnight in MRS-broth containing 10 μCi ^3^H-thymidine/ml for metabolic labeling, collected and washed twice with PBS. IPEC-1 cells grown on Thincert™ wells were washed once with PBS, and 125 μl of the labeled bacterial suspensions in DMEM/Ham’s F-12 [1:1] medium at A_600nm_ values of 0.25, 0.5 or 1 were added per well. The plates were incubated for one hour at +37°C, 5% CO_2_ followed by five washes with PBS. The cells were lysed by adding 250 μl of 1% SDS in 0.1 M NaOH per well and by incubating overnight at +37°C, and the radioactivity of the lysed samples (output) was measured by liquid scintillation counting. The input radioactivity values were determined by liquid scintillation counting of the cell suspensions in DMEM/Ham’s F-12 [1:1] (A_600nm_ = 0.25, 0.5 or 1), first treated with an equal volume of 1% SDS in 0.1 M NaOH overnight at +37°C. The adherence was expressed as the proportion (%) of the original radioactivity remaining, after first subtracting the background radioactivity from IPEC-1 cells incubated without bacteria (for outputs) and from DMEM/Ham’s F-12 [1:1] medium (for inputs).

### Inhibition of F4-fimbriated ETEC adherence to IPEC-1 cells by L*. amylovorus*

*L. amylovorus* cells were cultivated overnight, collected and washed twice with PBS. The F4^+^ ETEC strain was cultivated overnight in LB broth containing 10 μCi ^3^H-thymidine/ml for metabolic labeling. Labeled ETEC cells were collected and washed with PBS and the expression of F4 fimbriae was confirmed with the Fimbrex slide agglutination test kit (VLA Scientific, New Haw, UK). The inhibitory effects of the strains were tested in three different experimental set-ups: exclusion, competition and displacement. In each arrangement, 100 μl of *L. amylovorus* strains in DMEM/Ham’s F-12 [1:1] medium (A_600_ = 6) were added to IPEC-1 cells; in exclusion assays 1 hour before, in displacement assays 1 hour after, and in competition assays simultaneously with the addition of 100 μl of ^3^H –labeled ETEC in the same medium (A_600_ = 0.6). In the displacement assays, the unbound ETEC cells were removed by two washes with PBS before the addition of lactobacilli. The cells were further incubated for one hour at +37°C, 5% CO_2_, followed by five washes with PBS. Finally, the cells were lysed by adding 250 μl of 1% SDS in 0.1 M NaOH per well and by incubating overnight at +37°C, and the radioactivity of the lysed samples was measured by liquid scintillation counting. The proportion of adherent ETEC cells (%) in the presence or absence of the *L. amylovorus* strains was calculated as in the adhesion experiments, and the inhibition (%) was calculated according to the formula: [adherence (no La) – adherence (with La)] / adherence (no La) x 100%, where La indicates *L. amylovorus*.

### Growth inhibition of intestinal pathogens by the culture supernatants of *L. amylovorus*

The supernatants collected (650 g, 20 min, +4°C) from overnight cultures of the *L. amylovorus* strains were filter-sterilized through 0.22 μm pore-size filters and stored at -20°C. The inhibitory effects of the supernatants were assessed by monitoring the abilities of the pathogens to grow in the presence (10% V/V) of the supernatants in a microtiter plate format as previously described [36]. Briefly, the A_600nm_ values of the pathogen cultures were measured every 30 minutes with an automatic reader (Bioscreen C, Growth Curves Oy, Helsinki, Finland) in the presence or absence of pH-adjusted (pH 6.2) or non-adjusted culture supernatants at 36.5 +/-0.5°C, with three parallel wells for each supernatant and control. The inhibition was quantified using the area under the growth curve (AUC) obtained during the first 12 hours of growth, automatically created by the Research Express software (Transgalactic Ltd, Vantaa, Finland), and expressed as the area reduction percentage (ARP) as previously described [29]. Linear regression (SPSS) was used to estimate the relationship between the ARP values and colony forming unit (CFU) counts as previously described [36].

### Isolation and generation of human monocyte-derived dendritic cells (moDCs)

Leukocyte-rich buffy coats, donated by healthy volunteers, as well as the permission to use human leukocytes, were obtained from the Finnish Red Cross Blood Service. Monocytes were purified and cultured *in vitro* to generate moDCs using a method described earlier [37] with minor modifications. Briefly, peripheral blood mononuclear cells were first isolated by Ficoll-Paque (GE Healthcare, Little Chalfont, UK) density gradient centrifugation using Leucosep separation tubes (Greiner Bio-One, Germany), followed by a Percoll (GE Healthcare, Little Chalfont, UK) gradient centrifugation step. After magnetic beading using anti-CD3 and anti-CD19 beads (Dynal Invitrogen, Life Technologies, Carlsbad, CA), monocytes were allowed to adhere to 24-well plates (Falcon, BD, Franklin Lakes, New Jersey) for 1 h in the presence of RPMI 1640 (Sigma) supplemented with 20 mM HEPES, penicillin and streptomycin (100 IU/ml) and 2 mM L-glutamine without serum. The adhering cells were washed twice with PBS, after which differentiation was induced by maintaining the cells in RPMI 1640 (supplemented as described above) containing 10% (v/v) FCS (Integro, Zaandam, the Netherlands), 10 ng/ml human recombinant granulocyte macrophage-colony stimulating factor (GM-CSF, Gibco Life Technologies, Carlsbad, CA), and 20 ng/ml human recombinant interleukin 4 (IL-4, Gibco Life Technologies, Carlsbad, CA). MoDCs were used on day 7 in the experiments. In each experiment, cells from four donors were used.

### Stimulation of human moDCs and cytokine measurements

*L. amylovorus* strains were cultivated overnight, collected and washed with PBS. The A_600nm_ values of the bacterial suspensions were normalized, and the bacterial cells were added to human moDCs at the multiplicity of infection (MOI) 1, 10, and 100 in RPMI 1640 containing FCS, HEPES, antibiotics, and glutamine. The same medium without bacteria was used as a control. After 24 h, cell culture supernatants were collected and stored at -20°C before further analyses. The supernatants were analyzed with the Bio-Rad’s Bio-Plex Pro Cytokine assay using the Bio-Plex -200 platform (Bio-Rad, Hercules, CA). Human TNF-α, IL-1β, IL-6, IL-10, and IL-12 quantification was performed for undiluted samples according to the manufacturer’s instructions. Human IP-10/CXCL10 was measured separately with the OptEIA ELISA kit (BD, Franklin Lakes, New Jersey) using samples diluted with sample matrix RPMI 1640 medium.

### Purification of cell wall fragments (CWF) and coating of CWF by recombinant S-layer proteins

Cell wall fragments were purified from *L. amylovorus* cells as described earlier [33]. Purified cell walls were lyophilized and stored as suspensions in water at -20°C. In order to coat the cell walls, the affinity purified recombinant S-layer proteins were dissolved in 5 M GuHCl at a concentration of 30 μg/ml, dialyzed against 50 mM Tris–HCl (pH 7.0) at +4°C overnight and centrifuged (40,000 g, 30 min, +4°C) to remove large protein aggregates. The protein concentrations of the supernatants were determined by the Bradford method, immediately after which the supernatant proteins and the cell walls were combined in a ratio 1:4 (W/W) and incubated overnight at +4°C with rotation. The coated CWF were collected by centrifugation (25,000 g, 30 min, +4°C), resuspended into DMEM/Ham’s F-12 [1:1] medium and analyzed by SDS-PAGE. To verify the absence of large protein aggregates among the coated CWF, the preparations in 50 mM Tris–HCl (pH 7.0) were routinely negative stained by uranyl acetate (5 min on ice) and observed by JEOL 1200-EX II transmission electron microscope at the operating voltage of 80 kV.

### Adherence of S-layer protein-coated cell wall fragments to IPEC-1 cells

CWF to be used as uncoated controls were labeled by EZ-Link Sulfo-NHS-LC-Biotin (Thermo Scientific, Waltham, MA) according to the manufacturer’s instructions by adding 0.5 mg of label per 500 μg of CWF (dry weight). IPEC-1 cells grown on Thincert™ wells were washed once with PBS, and 80 μg of Slp-coated or uncoated CWF in the total volume of 100 μl DMEM/Ham’s F-12 [1:1] medium was added per well, corresponding to approximately 8 μg (0.13-0.18 nmol) of each S-layer protein per well containing 2.5 x 10^5^ IPEC-1 cells. The plate was incubated for two hours at +37°C and 5% CO_2_ followed by four washes with PBS. The cells were fixed with 4% paraformaldehyde (PFA) in PBS for 10 minutes at room temperature and washed three times with 0.1 M sodium phosphate buffer (pH 7.4). Slp-coated CWF were detected by an indirect immunofluorescence staining with Slp-specific immunoglobulins (20 μg/ml, purified by Hi Trap columns, GE Healthcare, Little Chalfont, UK) and AlexaFluor488-conjugated secondary antibodies (2 μg/ml, LifeTechnologies, Carlsbad, California), all in PBS-0.1% BSA, and uncoated cell walls were detected by staining with AlexaFluor488-conjugated streptavidin (2 μg/ml, LifeTechnologies, Carlsbad, California) in PBS-0.1% BSA. The bottoms of the Thincert™ wells were prepared for microscopy and observed in a Leica DM 4000B epifluorescence microscope (Leica Microsystems, Wetzlar, Germany). The mean number of adherent CWF was quantitated from 20 randomly selected fields of 3.5 x 10^4^ μm^2^, and representative photographs were taken with the Olympus DP70 digital camera system with the cell^P^ imaging software (Olympus Corp., Tokyo, Japan).

## Results

### Adherence of *L. amylovorus* strains to mucus

**Figure 1:**
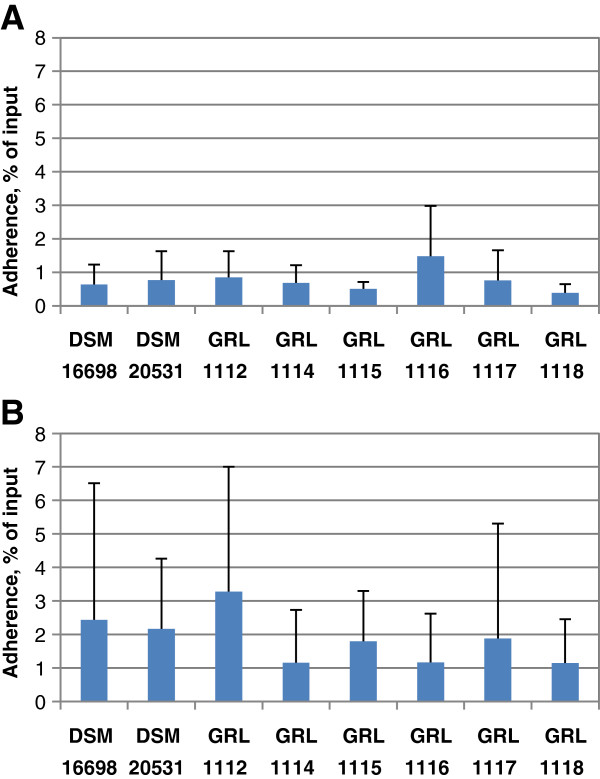
**Adherence of *L. amylovorus *strains to mucus.** The adherence of *L. amylovorus* strains to immobilized porcine gastric mucin (type II, Sigma) **(A)** and to mucus purified from porcine small intestine **(B)** was studied using bacterial cells labeled with the fluorescent dye SYTO^®^9. The means and standard deviations of 5–8 independent experiments are shown, each with three technical replicates.

The adherence of the *L. amylovorus* strains to commercially available porcine gastric mucins and to mucus isolated from the small intestine of a 8-week old pig were examined using *L. amylovorus* cells labeled by the DNA-binding stain SYTO^®^9 (Figure 1). None of the strains bound extensively to porcine gastric mucins, i.e., typically less than 1% of the original amount of cells remained mucin-bound (Figure 1A). The same was true for porcine intestinal mucus, where the proportion of adhering bacterial cells was usually 2% or less (Figure 1B). The very high variation between the experiments and the lack of any consistent dose–response of binding (data not shown) supported the conclusions.

### Adherence of *L. amylovorus* strains to IPEC-1 cells

**Figure 2:**
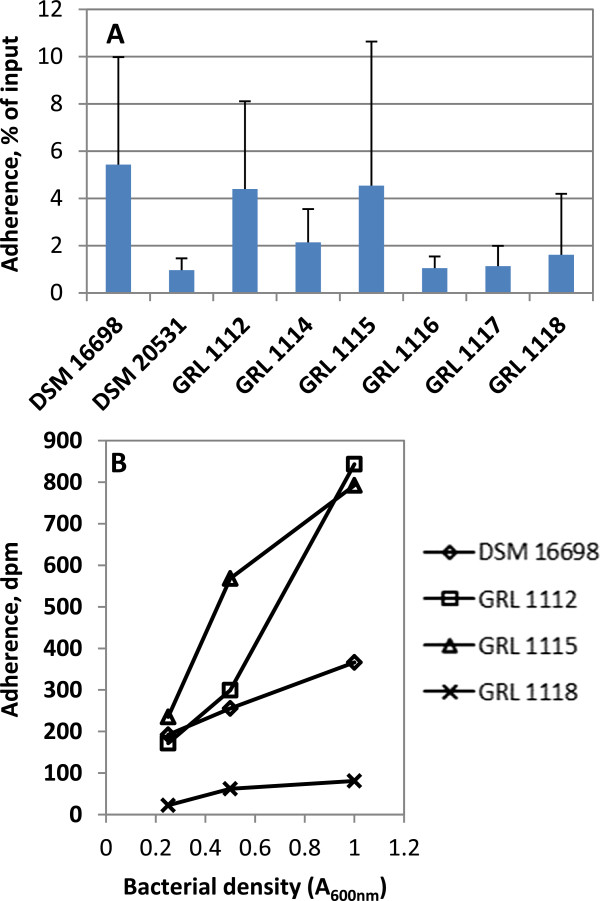
**Adherence of *L. amylovorus *strains to IPEC-1 cells. A)** The adherence of ^3^H-labeled *L. amylovorus* strains (A_600nm_ = 0.25) to IPEC-1 cells grown on Thincert™ wells showing the means and standard deviations of 3–7 independent experiments, each with three technical replicates. **B)** An example of the adherence of *L. amylovorus* DSM 16698, GRL 1112, GRL 1115 and GRL 1118 to IPEC-1 cells as the function of cell density. The means of three technical replicates from one representative experiment are shown. Dpm, disintegrations per minute.

In contrast to mucus binding, clear differences were observed in the adherence of the *L. amylovorus* strains to porcine small intestinal epithelial cells as represented by the cell line IPEC-1 (Figure 2A). The previously reported binding of *L. amylovorus* DSM 16698 to IPEC-1 cells [26] was confirmed, and the adherence of the strains GRL 1112 and GRL 1115 was found to be within a similar range, though the three strains displayed high variabilities in different experiments. In contrast, the type strain of *L. amylovorus*, DSM 20531^T^, isolated from silage, and the rest of the porcine intestinal isolates were clearly less adhesive. An example of the dose–response of binding is shown in Figure 2B.

### Inhibition of F4-fimbriated ETEC adherence to IPEC-1 cells by *L. amylovorus*

**Figure 3:**
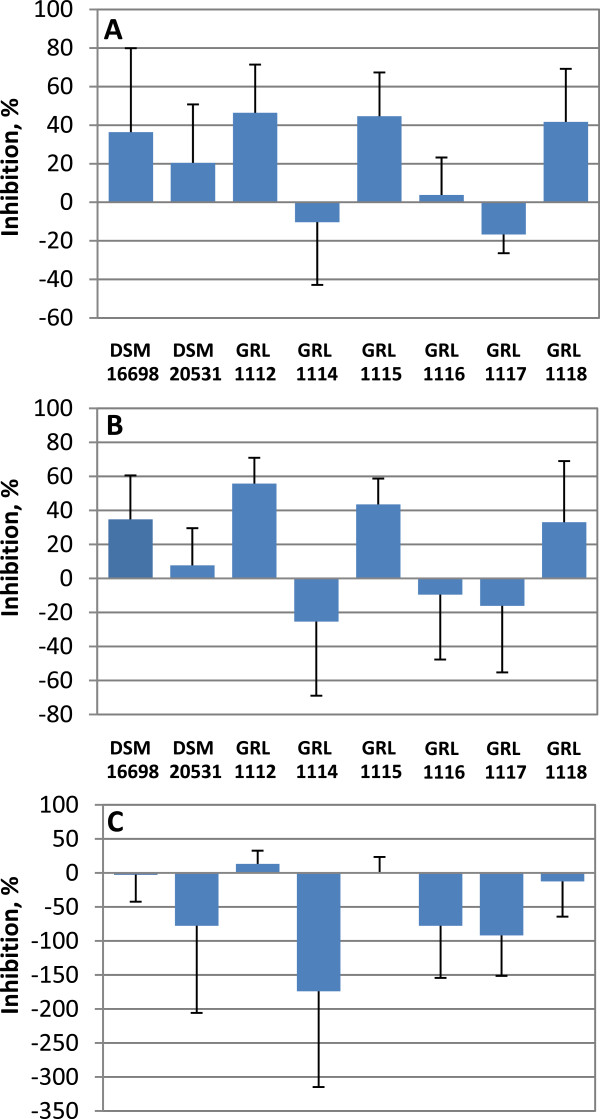
**Inhibition of F4-fimbriated ETEC adherence to IPEC-1 cells by *L. amylovorus.*** The inhibition of F4-fimbriated ETEC adherence to IPEC-1 cells by the indicated *L. amylovorus* strains in exclusion **(A)**, competition **(B)** and displacement assays **(C)** was tested with ^3^H-labeled ETEC cells as detailed in Methods. The means and standard deviations of 3–7 independent experiments are shown, each with three technical replicates.

IPEC-1 cells have been shown to support the adherence of *E. coli* carrying F4-type fimbriae [38]. Next the *L. amylovorus* strains were tested in three different experimental set-ups (exclusion, displacement and competition as described in Methods) to evaluate if the observed differences in their adherence to IPEC-1 cells correlated with their abilities to inhibit ETEC adherence in the same model. The results were evaluated by comparing the adherence of ETEC in the presence or absence of the *L. amylovorus* strains. The strains DSM 16698, GRL 1112, GRL 1115 and GRL 1118 were able to inhibit pathogen adherence if they were added beforehand (exclusion, Figure 3A) or simultaneously with ETEC (competition, Figure 3B); the strain DSM 20531^T^ achieved only a borderline inhibition when added beforehand (Figure 3A)*,* and the rest of the strains had a negligible or even a slightly enhancing effect on ETEC binding in both assays (Figures 3A and B). Importantly, none of the strains was able to displace previously bound ETEC from IPEC-1 cells (displacement, Figure 3C).

### Inhibition of pathogen growth by the culture supernatants of *L. amylovorus*

**Figure 4:**
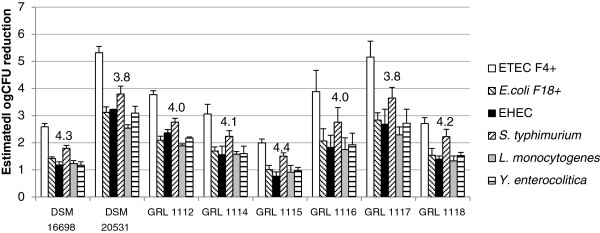
**Reductions in pathogen counts by *L. amylovous *culture supernatants.** Six different swine intestinal pathogens were grown in TSB medium in the presence of the filter-sterilized supernatants of the *L. amylovorus* strains, and the reductions in pathogen counts, expressed as log CFU values, were estimated from the area reduction percentages (ARPs) of the pathogen growth curves by linear regression. The average pH values of the supernatants are shown above the histograms. The results are the means and standard deviations of three independent experiments, each performed with fresh culture supernatants with three technical replicates.

The filter-sterilized culture supernatants of the *L. amylovorus* strains were assayed for their abilities to inhibit the growth of various intestinal pathogens (Figure 4). All the supernatants markedly inhibited the growth of the test pathogens. For instance, the supernatants of the strains DSM 20531^T^ and GRL 1117 reduced the growth of F4-fimbriated *E.coli* by more than 100 000-fold and the growth of *Salmonella typhimurium* almost by a factor of 10 000. The growth of F4-fimbriated *E. coli* was most efficiently inhibited. The pH values of the supernatants varied from 3.8 to 4.5. It is notable that the reductions in pathogen counts inversely correlated with the pH values of the supernatants (Figure 4), and culture supernatants which had been adjusted to the pH of plain MRS lowered the pathogen counts by much less than tenfold (data not shown), indicating that the inhibition was mainly due to the low pH associated with lactic acid production.

### Cytokine induction in moDCs by *L. amylovorus*

**Figure 5:**
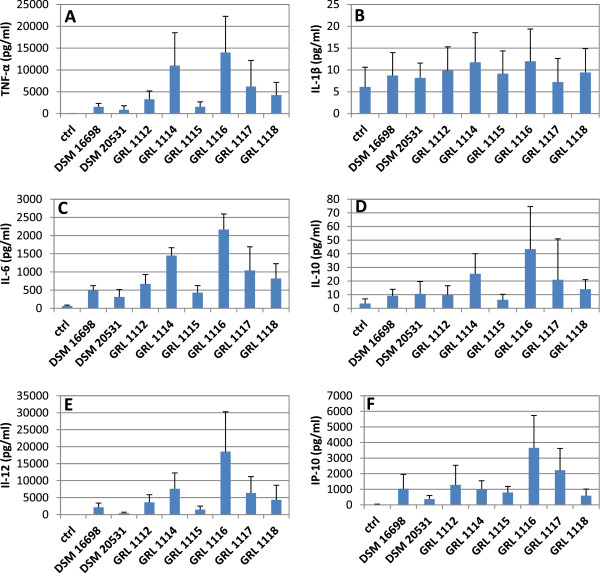
**Cytokine induction in human dendritic cells by *L. amylovorus.*** The extents of induction of TNF-α **(A)**, IL-1β **(B)**, IL-6 **(C)**, IL-10 **(D)**, IL-12 **(E)** and IP-10/CXCL10 **(F)** in human monocytic dendritic cells (moDCs) were tested after treating the cells with *L. amylovorus* strains for 24 hours at the bacteria/DC ratio 100:1. The data are presented as the means and standard deviations from one representative experiment out of three, performed with moDC:s of four donors.

The S-layer-carrying *L. acidophilus* strain NCFM interacts with human DCs eliciting an anti-inflammatory IL-10 response and it promotes the Th_2_-differentiation of T-cells through DC:s; the S-layer protein has been shown to have a role in this response [12]. Prompted by these findings, we examined the potential of the phylogenetically closely related, S-layer-carrying *L. amylovorus* strains to induce immune signaling in human DCs. As shown in Figure 5, when tested at the bacteria/DC ratio of 100:1, clear differences between the levels of cytokines induced by the strains were observed. Interestingly, the anti-inflammatory response induced by *L. acidophilus* NCFM was not observed with the *L. amylovorus* strains. Instead, our strains typically induced a mixed cytokine response with the release of both proinflammatory (TNF-α, IL-6, IL-1β, IL-12, IP-10/CXCL10) and anti-inflammatory (IL-10) cytokines from human DCs. Furthermore, the strain GRL 1116, which was most potent at inducing proinflammatory cytokines, induced also the highest levels of the anti-inflammatory cytokine IL-10. Analogously, the strain DSM 20531^T^ and GRL 1115 were among the weakest inducers of both pro-and anti-inflammatory cytokines. At the lower MOI values of 1 and 10, no clear induction of any of the cytokines was observed in comparison to the negative control (data not shown).

### Genomic characterization of *L. amylovorus* Slp:s

**Figure 6:**
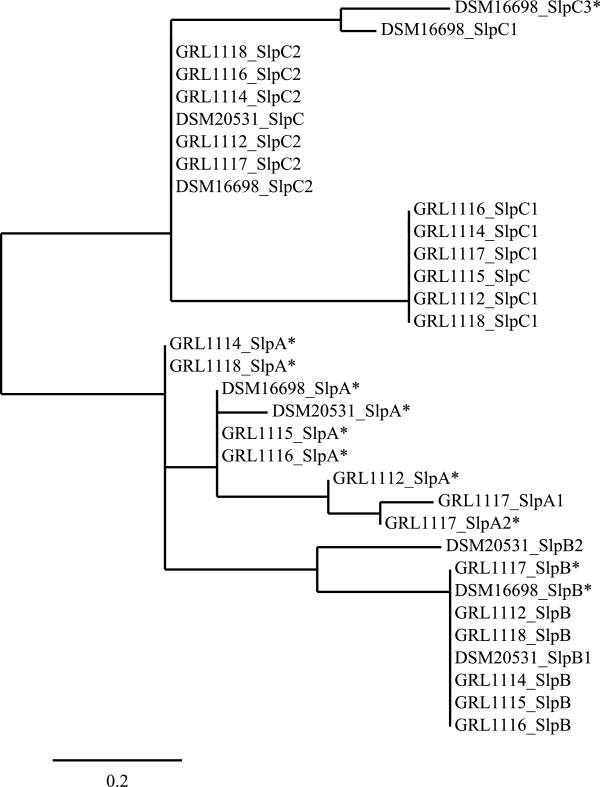
**Phylogeny of *L. amylovorus *Slp:s.** A neighbour-joining phylogenetic tree based on *L. amylovorus* Slp sequences was generated by creating a multiple amino acid sequence alignment of the predicted S-layer proteins with MUSCLE [39], by eliminating poorly aligned positions using GBLOCKS [40], and by generating phylogenies using the PhyML package [41]. Numbers 1–3 indicate the presence of several *slp* genes in the same strain. *, the corresponding gene is expressed.

To initiate comparative studies on the role of *L. amylovorus* surface layer proteins in the probiotic interactions described above, the numbers and sequence similarities of the *slp* genes in the genomes of the strains were initially analysed. The genomic investigation of the eight strains revealed several *slp* genes in each strain. Genes with homology to *L. acidophilus* NCFM *slpA* and *slpB*[12] were identified, and the homologous *L. amylovorus* genes were named *slpA* and *slpB*, respectively. Furthermore, *slp*-like genes of a third type were detected in all of the eight genomes and these were designated as *slpC*. The *slp* sequences, along with the deduced amino acid sequences, are shown in Additional file 1. All the eight strains studied carried only one *slpA*-homologue, except for GRL 1117, which had two distinct *slpA*-like genes (*slpA1* and *slpA2*). Only one *slpB*-homologue was identified in GRL 1112, 1114, 1115, 1116, 1117 and 1118 as well as in DSM 16698, whereas DSM 20531^T^ carried two *slpB*-like genes (*slpB1* and *slpB2*). The highest variation was found in the number of *slpC*-type genes: strains DSM 20531^T^ and GRL 1115 carried one, DSM 16698 possessed three (*slpC1*, *slpC2*, *slpC3*), and the rest of the strains had two *slpC*-type genes (*slpC1* and *slpC2*). Exceptionally, the gene *slpC3* of DSM 16698 was found to be located on a plasmid. A phylogenetic tree was constructed based on the deduced amino acid sequences of the *slpA*, *slpB* and *slpC* gene products (Figure 6). The tree clearly shows that the SlpA-like sequences have diversified most during evolution, while the SlpB-type proteins have remained more similar to each other whereas the predicted SlpC-type proteins could be clustered into three distinct groups.

### Expression analysis of *slp* genes and comparison of surface-located Slp:s *in silico*

**Figure 7:**
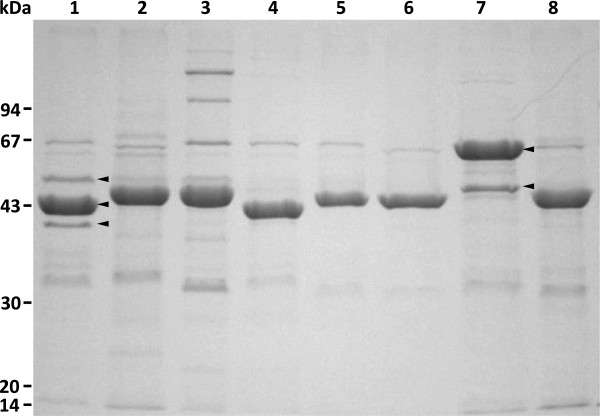
**SDS-PAGE analysis of *L. amylovorus *strains.** Intact cells of *L. amylovorus* DSM 16698 (lane 1), DSM 20531^T^ (lane 2), GRL 1112 (lane 3), GRL 1114 (lane 4), GRL 1115 (lane 5), GRL 1116 (lane 6), GRL 1117 (lane 7) and GRL 1118 (lane 8) from 50 μl of overnight cultures (A_600nm_ = 6.4) were boiled in standard Laemmli sample buffer (extracting surface proteins) and the supernatants were analyzed by standard SDS-PAGE in a 12% gel. Arrowheads indicate SlpA (44 kDa), SlpB (50 kDa) and SlpC (40 kDa) of *L. amylovorus* DSM 16698 (lane 1) and SlpA (61 kDa) and B (49 kDa) of GRL 1117 (lane 7).

In an attempt to reveal which of the identified *slp* genes encoded the S-layer protein bands seen in the surface protein profiles of the strains (Figure 7), either an amino-terminal sequencing or a peptide mapping analysis was performed for the proteins, and the results were compared with the genomic sequence data. In this study, the major S-layer protein bands of the *L. amylovorus* isolates GRL 1112-GRL 1118 [28] were all shown to be encoded by *slpA*-like genes. The surface protein profiles of the strains DSM 16698 and DSM 20531^T^ also revealed one major protein band, approximately 45 kDa in size (Figure 7), and, based on N-terminal sequencing, this represented the protein encoded by *slpA*. The presence of an S-layer on the surface of *L. amylovorus* DSM 16698 and DSM 20531^T^ was thus confirmed in this study. Furthermore, the two additional surface protein bands of DSM 16698, approximately 50 kDa and 40 kDa in size, were found to represent the products of *slpB-* and *slpC-*like sequences, respectively. Of the three *slpC*-type genes present in the DSM 16698 genome, the plasmid-borne version, *slpC3*, was found to be expressed. Despite the presence of the SlpC-encoding gene on a plasmid, the SlpC band was invariably present in the SDS-PAGE profile of DSM 16698. In indirect immunofluorescence assays, SlpA and SlpB of DSM 16698 were identified on the bacterial surface as predicted. In contrast, SlpC remained undetectable, suggesting that the location of SlpC is not accessible to antibodies due to shielding by other cell envelope components (data not shown). The expressed *slp* genes of the *L. amylovorus* strains are highlighted in Figure 6.

The designations and calculated molecular weights of the S-layer proteins present on the bacterial surface are summarized in Table 1, and the deduced amino acid sequences of these proteins are found in Additional file 1. The analysis of the Slp amino acid sequences revealed the typical features of *Lactobacillus* S-layer proteins, including a high predicted pI value (9.1-9.6) and a very low proportion of sulfur-containing amino acids [8]. A pairwise comparison of the amino acid sequence similarities of these proteins is shown in Table 2. An amino acid sequence alignment of these Slp:s and the major, surface-located S-layer proteins of *L. acidophilus* NCFM (SlpA, GenBank AAV42070) and *L. crispatus* JCM 5810 (CbsA, GenBank AF001313) is shown in Additional file 2. All the *L. amylovorus* S-layer proteins, with the exception of SlpC, display significant overall similarity to the *L. acidophilus* NCFM and *L. crispatus* Slp:s, with the signal peptides and the carboxy-terminal thirds of the sequences being particularly well conserved.

**Table 2:** **Amino acid sequence similarities between *L. amylovorus *** **S-layer proteins present on the bacterial surface**

	**DSM16698_SlpA**	**DSM16698_SlpB**	**DSM16698_SlpC3**	**DSM20531_SlpA**	**GRL1112_SlpA**	**GRL1114_SlpA**	**GRL1115_SlpA**	**GRL1116_SlpA**	**GRL1117_SlpA2**	**GRL1117_SlpB**	**GRL1118_SlpA**
**DSM16698_SlpA**	100^1^										
**DSM16698_SlpB**	26.8	100									
**DSM16698_SlpC3**	12.5	16.9	100								
**DSM20531_SlpA**	48.2	22.7	15.1	100							
**GRL1112_SlpA**	36.1	25.4	13.0	34.8	100						
**GRL1114_SlpA**	51.7	27.2	14.1	53.6	36.9	100					
**GRL1115_SlpA**	53.7	21.1	14.1	55.0	38.6	48.9	100				
**GRL1116_SlpA**	53.1	21.5	13.3	53.2	38.6	48.4	95.5	100			
**GRL1117_SlpA2**	37.0	20.0	12.5	37.3	47.8	32.1	34.5	32.6	100		
**GRL1117_SlpB**	24.0	69.6	17.1	20.2	21.7	22.6	21.2	21.2	22.0	100	
**GRL1118_SlpA**	49.4	23.3	12.5	53.6	36.0	48.8	54.2	55.8	35.0	18.9	100

^1^Pairwise scores were calculated for each pair of sequences by calculating the number of identities in the best CLUSTALW alignment, and by dividing by the number of residues compared (gap positions were excluded).

### The role of S-layer proteins in adherence to IPEC-1 cells

**Figure 8:**
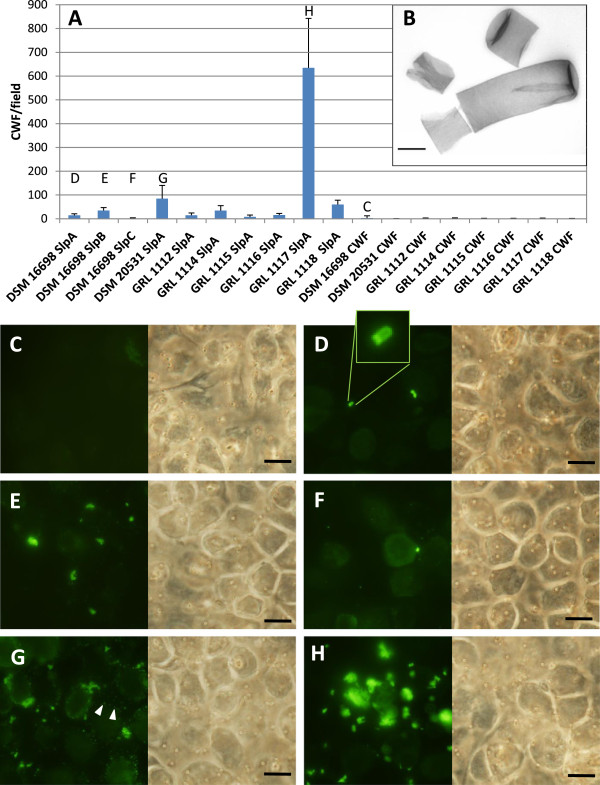
**Adherence of S-layer protein-coated cell wall fragments to IPEC-1 cells. A)** The IPEC-1 cell adherence of CWF, coated or uncoated by the indicated S-layer proteins, expressed by quantitative means. The mean number of adherent CWF was quantitated from 20 randomly selected fields of 3.5 x 10^4^ μm^2^ and the results are presented as means and standard deviations from one representative experiment out of three; letters above the bars refer to Figures C-H below. **B)** An electron micrograph of purified CWF of *L. amylovorus* DSM 16698. Scale bar, 0.5 μm. **C-H)** Examples of the adherence of Slp-coated and uncoated *L. amylovorus* CWF to IPEC-1 cells as detected by fluorescence. The figures show the adherence of uncoated *L. amylovorus* DSM 16698 CWF **(C)** and the adherence of the following Slp/CWF complexes: DSM 16698 CWF/SlpA **(D)**, DSM 16698 CWF/SlpB **(E)**, DSM 16698 CWF/SlpC **(F)**, DSM 20531^T^ CWF/SlpA **(G)** and GRL 1117 CWF/SlpA **(H)**. The rightmost figures display the corresponding fields viewed with phase contrast optics. The inset in **(D)** shows a magnified image of a cell wall fragment. Arrowheads in **(G)** indicate precipitated Slp. Scale bars, 10 μm.

The poor water-solubility of *Lactobacillus* S-layer proteins, resulting from the inherent self-assembly property of bacterial S-layers *in vitro*, sets limitations on what methods can be used to assess the adherence of S-layer proteins to a particular target. In order to avoid potential unspecific effects associated with protein precipitation in adhesion experiments, a protein presentation system, based on purified *L. amylovorus* cell wall fragments as S-layer protein carriers, was developed and used to study the role of the surface-located *L. amylovorus* Slp:s in adhering to IPEC-1 cells (see Figure 8B for an electron micrograph of purified CWF). This method is based on the inherent tendency of S-layer proteins to recrystallize in a native manner on CWF [42,43], which have been purified in such a way to remove all of the non-covalently attached components (including the endogenous S-layer proteins), but preserving the covalently attached polymeric components like teichoic acids and polysaccharides, thus ensuring the proper self-assembly of the recombinant Slp:s. However, purified cell wall fragments are of low density and have poor contrast, necessitating specific staining if one wishes to detect the CWF on epithelial cells. For Slp-coated CWF, an indirect immunofluorescence staining procedure with Slp-specific antibodies was used, but as we failed to obtain functional antibodies against purified cell wall fragments (data not shown), the detection of uncoated control CWF was based on their prior biotinylation and staining with labeled streptavidin after the adherence assay.

Figure 8A shows the adherence of CWF, coated or uncoated by *L. amylovorus* cell surface-located Slp:s, to IPEC-1 cells. In Figures 8C-H, micrographs illustrating the results of the binding assay in (A) are shown. The adherence of all uncoated CWF was negligible, as exemplified by the adherence of the CWF of the strain DSM 16698 in Figure 8C. The major Slp:s of the *L. amylovorus* strains DSM 16698 (Figure 8D), GRL 1112 and GRL 1115 adhered poorly to IPEC-1 cells, although the intact cells of these strains were adhesive (Figure 2). The minor S-layer like protein SlpB of DSM 16698 exhibited some adhesiveness (Figure 8E), when compared to SlpA (Figure 8D) or SlpC (Figure 8 F) of the same strain. Surprisingly, the S-layer protein SlpA of the weakly adhering strain GRL 1117 (Figure 8H) and, to a lesser extent, the Slp:s of some of the other weakly adhering strains, e.g. DSM 20531^T^ (Figure 8G) and GRL 1118 also displayed affinity for IPEC-1 cells.As detailed in Methods, special care was taken to minimize the formation of S-layer protein precipitates during the coating procedure of CWF. However, the presence of small Slp aggregates, as indicated by the small, dot-like, immunoreactive material among the coated cell walls, could not be completely avoided (see Figure 8G as an example). However, the quantification of the result by microscopic counting made it possible to ignore this undesirable signal, probably originating from unspecific and/or irrelevant binding.

## Discussion

In this study, seven porcine intestinal *L. amylovorus* strains, and the type strain of *L. amylovorus*, which is not of intestinal origin, were characterized *in vitro* for their abilities 1) to adhere to porcine mucus 2) to bind to epithelial cells of the pig small intestine, 3) to inhibit the adherence of an F4-fimbriated ETEC strain to porcine intestinal epithelial cells, 4) to produce soluble inhibitors against intestinal pathogens and 5) to induce immune signaling in dendritic cells.

None of the eight strains studied exhibited any efficient adherence to porcine gastric or intestinal mucus, as the level of adherence was around 2% or less. In previous studies, lactic acid bacteria or pathogens exhibiting similar levels of adhesion have been among the least adhesive strains, and they are considered to be non-adhering [35,44,45]. In addition, the highly variable adherence of our strains in the different experiments strongly suggests that the binding was non-specific e.g. being due to hydrophobic interactions, a common complication encountered in interpreting the results of mucus binding studies [46,47]. Furthermore, the analysis of the genomic sequences of *L. amylovorus* GRL 1115 and GRL 1116 revealed the complete absence of genes encoding putative mucus binding proteins in these strains (unpublished). Although the presence of unidentified mucus adhesins cannot be completely excluded, the level of adherence of these strains to mucus can be considered as negligible. Both efficiently and poorly mucus-binding *Lactobacillus* strains have been isolated from the intestine and milk of swine [48-51], and the lack of a mucus-adhering capability is not uncommon among the widely used human probiotic lactobacilli [52]. Furthermore, growth conditions not tested in this study, such as cultivation on a solid medium or the addition of mucin to the standard culture, might have triggered the mucus-binding capacities of the strains, as described previously for *L. reuteri*[48]. Other binding functions, such as the capacity to adhere to extracellular matrix components, have previously been described for these GRL-strains [28].

The adherence of the *L. amylovorus* strain DSM 16698 to IPEC-1 cells and its ability to inhibit the binding of ETEC to IPEC-1 cells in a competition-type assay have been demonstrated previously [26]. In this study, the reported adhesion of DSM 16698 to IPEC-1 cells was confirmed, and the adherence of GRL 1112 and 1115 was found to be at a similar level. These three well-adhering strains were also able to inhibit the adherence of ETEC to IPEC-1 cells in competition and exclusion assays. Surprisingly, the poorly adhesive strain GRL 1118 similarly inhibited ETEC adherence, suggesting that mechanisms other than competitive binding were involved in the inhibition, e.g., secreted inhibitory factors or coaggregation with the pathogen [53]. However, the spent culture supernatant of GRL 1118 did not reduce the growth of F4-positive ETEC more than the culture supernatants of the other GRL strains, and the growth inhibition in all cases was mainly attributable to the production of lactic acid. The production of substances specifically able to inhibit adherence was not tested in this study. Thus, at present we have no clear explanation for the observed inhibition of ETEC binding to IPEC-1 cells by GRL 1118.

One of the main mechanisms of probiotic action in the gastrointestinal tract is the modulation of mucosal and systemic immune responses [54]. These immunomodulatory properties, including immunoregulatory and tolerance-promoting, as well as pro-inflammatory functions, have been suggested to result from the stimulation of mucosal dendritic cells by probiotic bacteria [55,56]. Many different *Lactobacillus* species have been shown to modulate dendritic cell responses in studies with human or murine DCs [12,55-63]. *L. reuteri* ASM 20016, *L. casei* NIZO B255 [55] and *L. acidophilus* NCFM [12] specifically bind the DC-SIGN molecule (**d**endritic **c**ell **s**pecific C-type lectin **i**ntercellular adhesion molecule 3-**g**rabbing **n**on-integrin) on dendritic cells, triggering the differentiation of naïve T cells towards the T_reg_[55] or Th_2_[12] functional types. The bacterial component of *L. acidophilus* NCFM which interacts with DC-SIGN is its S-layer protein SlpA [12]. However, proinflammatory or Th_1_-polarising effects in DCs have also been described for NCFM [64-66]. These responses have been attributed to either lipoteichoic acid [66] or the S-layer associated protein encoded by the gene in locus Lba-1029 of NCFM [67]. *L. amylovorus* and *L. acidophilus* are phylogenetically closely related [68], and the S-layer protein of NCFM shows remarkable amino acid sequence similarity with the major Slp:s of the *L. amylovorus* strains studied (see Additional file 2). These findings led us to investigate the potential of our S-layer-carrying *L. amylovorus* isolates to induce cytokine production in human DCs. There were evident strain-specific differences in *L. amylovorus* immunomodulating capacities, but no clear preference was noted for any of the strains for inducing cytokines to drive the immune response exclusively towards either the Th_1_ or the Th_2_ type. Instead, most of the strains induced both pro-inflammatory cytokines (TNF-α and IL-6), IL-12 favouring a Th_1_ response and IL-10 favouring a Th_2_-type response, a phenomenon that has also been demonstrated for *L. gasseri* strains [63]. Although the specific immunomodulating surface molecules of *L. amylovorus* remained unidentified, the results of this work emphasize the importance of strain-specific differences in the immunomodulating capacities and are thus in line with previous studies [57,59,61,63]. Considering the probiotic potential of lactobacilli, it is clear that for the optimal performance of the complex immune system, Th_1_, Th_2_ and T_reg_ responses have to be balanced. The most successful manipulation by probiotics will also depend on the dose and strain combination of probiotic bacteria, the type of pathogen challenge, and the specific environmental conditions [55,57,69].

In the *in silico* analysis of the *L. amylovorus* S-layer proteins, we found that the amino acid sequences of the *L. amylovorus* Slp:s studied, excluding SlpC, were very similar to the amino acid sequence of *L. acidophilus* NCFM SlpA, especially in the carboxy-terminal region, a phenomenon observed among the S-layer proteins of other *L. acidophilus*-related lactobacilli as well [70]. The pattern of conservation apparently reflects the well-known role of the carboxy-terminal domains in cell wall binding [70,71], and strongly suggests that the cell-wall binding function also resides in the carboxy-terminal region in *L. amylovorus* Slp:s. The amino-terminal parts of *L. amylovorus* Slp:s, apparently facing the environment, are more variable, but the valine-rich regions, which flank the amino-terminal domain in the S-layer protein CbsA of *L. crispatus*, and which have been shown to be important for the self-assembly of CbsA monomers [70], were however conserved in most of the studied *L. amylovorus* S-layer proteins.

S-layers typically form the outermost layer of the bacterial cell, making them attractive candidates for being involved in adherence to host cells. However, attempts to create completely S-layer negative *Lactobacillus* mutants have been unsuccessful [11,72-74], emphasizing the necessity of at least one functional S-layer protein for lactobacilli and compelling us to investigate the role of these proteins by utilizing protein-level methods rather than with knock-out mutants. However, as Slp:s are poorly soluble, the presence of S-layer proteins in an aggregated form in the assays may evoke unspecific effects and compromise the reliability of the results. Indeed, due to the methodological difficulties related to the poor water-solubility of S-layer proteins, the results of most of the previous reports examining the role for *Lactobacillus* S-layers in adherence have remained ambiguous, and so far only a few *Lactobacillus* S-layer proteins have been convincingly shown to act as epithelial cell adhesins or to bind to extracellular matrix proteins or immune cells [9-13]. In our experiments, we created a protein presentation system based on the self-assembly of recombinant *L. amylovorus* S-layer proteins on purified cell wall fragments from each strain. The method of coating the CWF was designed to minimize Slp precipitation being based on the finding that even in an aqueous buffer, a small fraction of Slp molecules remains visibly unprecipitated, and this dilute protein fraction can be separated from the precipitate by centrifugation, as previously described [33]. In the carrier system, the CWF-protein complexes are handled similarly as whole bacterial cells and thus the method largely evades the solubility-related problems. Furthermore, it allowed the use of uncoated CWF as controls and the presentation of the proteins in the native, symmetric organization observed on the bacterial surface, i.e. the obtained results possess real biological relevance.

The results of the adhesion experiments clearly indicated that none of the major S-layer proteins of the *L. amylovorus* strains on their own mediated bacterial adherence to IPEC-1 cells: when compared to uncoated cell walls, all the proteins exhibited at least a low level of adherence irrespective of the adhesive capacity of the bacterial strain from which the protein had originated. However, the putative co-operative role in adherence of other non-covalently attached cell wall components (e.g. Slp-associated proteins), removed during the preparation of CWF, cannot be completely excluded. On the other hand, the finding that the S-layer protein of the poorly adherent strain GRL 1117 bound highly efficiently to IPEC-1 cells suggests that some component(s) on GRL 1117 shield(s) the S-layer proteins, preventing them from interacting with IPEC-1 cells. An analogous phenomenon has been observed in *Lactobacillus rhamnosus* GG: the exopolysaccharide component shields the mucus-binding fimbriae, reducing the adhesive capacity of the strain for mucus [75,76]. Genes putatively participating in exopolysaccharide synthesis have been identified in all of the *L. amylovorus* strains studied (unpublished), but so far no biochemical evidence of their presence has been described.

## Conclusions

The swine intestinal *L. amylovorus* strains investigated in this study varied in their abilities to adhere to the porcine small intestinal epithelial cell line IPEC-1, while none of the strains adhered efficiently to porcine gastric or intestinal mucus. Several of the strains markedly inhibited the adherence of F4-fimbriated ETEC to IPEC-1 cells, and all inhibited the growth of various intestinal pathogens *in vitro*. The abilities of the strains to adhere to IPEC-1 cells were often associated with, but were not necessary for, the exclusion of F4-fimbriated ETEC from these cells, suggesting that additional mechanisms, other than competitive binding, were involved in the inhibition. The major S-layer proteins of the strains alone did not mediate the adherence of the strains to IPEC-1 cells. The immunological responses induced in human dendritic cells by the strains were of varying intensity and of a mixed type with both pro- and anti-inflammatory cytokines induced, with the same strain being the most potent inducer of both types of cytokines. The results indicate that while some commensals show adhesive capacity to epithelial cells, many may co-exist and benefit the host by mechanisms not attributable to adhesion to epithelial cells or mucus. The results warrant further studies of these swine intestinal strains if they are to be developed as probiotic feed supplements.

## Abbreviations

CWF: Cell wall fragments; DC: Dendritic cell; DMEM: Dulbecco’s modified eagle medium; EHEC: Enterohaemorrhagic *Escherichia coli*; ETEC: Enterotoxigenic *Escherichia coli*; F-12: F-12 Nutrient Mixture; FCS: Fetal calf serum; GuHCl: Guanidine hydrochloride; HEPES: 4-(2-hydroxyethyl)-1-piperazineethanesulfonic acid; moDC: Monocyte-derived dendritic cell; MOI: Multiplicity of infection; MRS: de Man-Rogosa-Sharpe; PBS: Phosphate buffered saline; PMSF: Phenylmethylsulfonyl fluoride; S-layer: Surface layer; Slp: Surface layer protein.

## Competing interests

The authors declare that they have no competing interests.

## Authors’ contributions

UH planned and performed most of the experiments and prepared the manuscript. RK was responsible for the *in silico* genomic analyses, TEP for the immunological and TL for the growth inhibition experiments; RK, TEP and TL also wrote sections of the manuscript. KH and JB participated in performing bacterial adhesion and ETEC binding inhibition experiments, HS had an accessory role in the management of the genomic data. SÅJ participated in the detection and expression analysis of *slp* genes and performed approximately half of the cloning and heterologous expression work. AP supervised and designed the study and participated in writing. All authors read and approved the final manuscript.

## Supplementary Material

Additional file 1 ***L. amylovorus slp *sequences.** A .docx-file showing the nucleotide and deduced amino acid sequences of the *slp* genes of the *L. amylovorus* strains studied. Click here for file

Additional file 2 **Amino acid sequence alignment of *L. amylovorus*, *L. acidophilus *and *L. crispatus *S-layer proteins.** A .tiff-image showing the alignment of the amino acid sequences of the surface-located Slp:s of the *L. amylovorus* strains studied, SlpA of *L. acidophilus* NCFM (LA, GenBank AAV42070) and CbsA of *L. crispatus* JCM 5810 (LC, GenBank AF001313), aligned by ClustalW available at http://www.ebi.ac.uk/Tools/msa/clustalw2/. Click here for file
