# Supplementary material for: Functional characterization of probiotic surface layer protein-carrying Lactobacillus amylovorus strains
Source: BMC Microbiol. 2014 Jul 28;14:199. doi: 10.1186/1471-2180-14-199 (PMC4236617; doi:10.1186/1471-2180-14-199)
Supplement: Additional file 1 — L. amylovorus slp sequences. A .docx-file showing the nucleotide and deduced amino acid sequences of the slp genes of the L. amylovorus strains studied. [file 1471-2180-14-199-S1.docx]

>DSM16698_SlpC1

MKKNKIILASVAALMAVSPMLSFGSQAHTVQAADTNDTNKMIMHTAVAYD

KDGNSTGVKYNAFSYARLVAGPVKIGDGIYYKVADKDQYLKATNIDGVTR

RITHNTYIYSTSTRRTSYQNRWKLYRGQTITTYGGSYRFKNGKHCFRVGG

PAKQYVKSYNLGPVIRANTTMAPNSNNTSSNTTTNKPTDNTTTNNTQPDE

TNVTVTARVANLCVEVPDKDMVQPSGKTAKMGDKFVVDRLEQGTRAGTGR

DGDDDWELAIYHIKGTNYWIYNYAVKAAKDIYHADGTVINFNGDRPRKQS

GRFKVDKLLYLWVPSENKAELFYHLVYNSVEGSKGSLNFTDGYVKASDVK

FDTDSVALTPSNTAAEAEAAAKK

>DSM16698_*slpC1*

atgaagaaaaacaagattatattagcctcagttgcagctttaatggctgt

aagtccaatgctttcatttggctcacaggctcacacagttcaagctgcag

ataccaatgatacaaataagatgattatgcacaccgctgttgcttatgat

aaagatggcaacagcactggcgtaaaatataatgcatttagctatgctag

attagtagctggccctgtaaagattggtgatggcatttattataaagttg

ccgataaagatcaatatttaaaggctactaacattgacggtgtaactaga

agaattacacataacacttacatctacagtacttctacacgtcgtactag

ttaccaaaatagatggaagttgtacagaggtcagacaatcactacttatg

gtggttcttacagatttaagaatggcaagcactgcttcagagtaggcggt

ccagctaagcaatacgttaaatcatataacttaggaccagttattagagc

caacaccacaatggctccaaatagcaataatacttcatcaaataccacta

ctaacaagccaactgacaacacaacaaccaataatactcaaccagatgaa

actaatgtaactgtaactgctagagttgccaacctttgtgtagaagtacc

tgataaagatatggtccaaccttctggaaagacagctaaaatgggagata

agtttgtagttgaccgcctagaacaaggaacacgtgctggtactggacga

gacggtgatgatgactgggaattagccatttaccatatcaaaggtacaaa

ctactggatttataattatgctgttaaagcagctaaagatatttaccatg

ctgacggtactgtaattaactttaatggtgatcgccctcggaaacaatct

ggacgttttaaggttgataaattactttatctttgggttccaagtgaaaa

caaagcagaattattctatcatttagtttataattctgtagaaggtagca

agggtagccttaactttactgatggctatgtaaaagctagtgatgttaaa

tttgacactgacagcgttgctttaacaccatcaaacacagctgctgaagc

agaagctgccgcaaagaagtaa

>DSM16698_SlpC2

MKKNKLILASVAALMAVSPVLSFGSQTHTVQAADTTITKTIMHTSMAYDR

DGKSTGTKYYAYKTVDVMTKPVKINDNLYYKVNGLNHYLRATNIDGVTRK

VTHNTYVYKSSNGRTSYNGRWKLYKGETVTTYGGFYKFKNGKLYFRIGGP

SKQYIKSANLGPVIKINTSVNGSSSSSTSTNLEETTVTVTTPTRLITQTS

NGYKGTAHITPVGTKFTVDRLEFNELSKRSENDDHFYHIKGTNQWINASD

VKAAKSIPLHDYFFENFSYITFPKDTDVYNADGTIQDHNGQKISKQKGQL

KVDKLVYIWVPSENKAELFYHLVGTSFYASTTPTVHWSTINVGHNAYVKA

SDVKFIDGSVKLTPSNTAAEAEAAAKK

>DSM16698_*slpC2*

atgaagaaaaacaaacttatcttagcttcagttgcggctttgatggctgt

aagcccggtgctttcatttggctcacagactcacacagttcaagctgcag

acactactattactaaaacaatcatgcatacttcaatggcatatgacaga

gatggtaagagcacaggtaccaaatattatgcatacaagaccgttgatgt

aatgactaagcctgtaaaaataaatgacaatttgtattacaaagtaaatg

gactaaatcactatttaagagctaccaatattgatggtgtaacgcgtaaa

gtaacgcataacacttatgtttataagtcttccaatggtcgtacttcgta

taacggcagatggaagctttataaaggtgaaacagtcactacttatggtg

gtttttacaaattcaaaaatggcaagctctacttcagaattggtggtccc

agcaaacaatacattaaatcagcaaatttaggaccagttattaaaattaa

tacttcagtgaatggatctagttcatcatcaacatctactaatttggaag

aaacaacagtcactgtaaccactccaactcgtttaattacccaaactagt

aatggctataagggaacggctcatattactccggtaggaacaaaatttac

agttgatcgtttggaatttaatgaactttctaagagaagtgaaaacgacg

accatttttatcatatcaaaggtactaatcagtggattaatgcttctgat

gtgaaagctgctaagagtattccactacatgactatttctttgaaaactt

ctcatacattacattccctaaagatactgatgtgtataatgcagacggaa

ctatccaagatcataacggtcaaaaaataagtaaacaaaaaggccaattg

aaagtagacaagttagtatatatttgggtgccaagtgaaaataaggctga

attattctatcatttggtaggtacaagcttttatgcaagtacaacgccaa

ccgttcattggagcacaattaatgttgggcataatgcttatgtaaaagcc

agcgatgttaagtttattgacggaagcgtaaagctaactccatcaaacac

agccgctgaagcagaagctgctgcgaagaagtaa

>DSM16698_SlpC3

MRKNKFILASVAALMAVSPMLPLSSQAHTVQAADTNAVNKMVMHTAVAYD

KDGNSTGVKYNAFSYARLLSTPVKIDGTIYYKVADKDQYLKATNIDGVTR

RITHNTYIYRTSTRRTSYQNRWKLYKGQTITTYGGSYRFKNGKHYFRVGG

PAKQYVKSYNLGPVIRANTTMAPNSNNTSSNTTTNKPTDNTTTNNTQPVG

NEETTVTVTASFNVNIFDNQGNTVRRNVPKGTKFVVDRLEVTPFADRFPT

ALGREGLYRIKGTDTWILAVDVTAAKKLPLHDYDLEHNSYIKFPQATDLY

NASGSKINTNGDYIRKQSGQYKVDKLLYIWVPSENKAEPFYHLVGKEVAS

NNGHISFDDGYVKASDVQFMSNSKAITPSNTAAEAEAAAKK

>DSM16698_*slpC3*

atgagaaaaaacaaatttatcttagcctcagttgcagctttaatggctgt

aagcccaatgctaccactcagttcacaggctcacacagttcaagctgcag

atactaatgctgtaaacaaaatggttatgcacactgctgttgcttatgat

aaagacggtaacagcactggcgtaaaatacaatgcatttagttatgctag

attgctatctacacctgtaaaaattgatggcactatttactataaagttg

ccgacaaagatcaatatttaaaggctaccaacattgatggtgtaactaga

agaattacccacaacacttacatctatagaacctctactcgtcgtactag

ttaccaaaatagatggaagttatataaaggacaaacaatcactacctacg

gtggttcttacagattcaagaatggtaagcattacttcagagtaggcggt

ccagctaagcaatatgttaaatcatataacttaggaccagttattagagc

caacaccacaatggcaccaaatagcaataatacttcatcaaataccacta

ctaataaaccaactgacaacacaacaaccaataatactcaaccagtaggt

aatgaagaaaccactgtaactgtaactgcttcattcaacgtaaatatctt

tgataatcagggtaacacagtaagaagaaacgttccaaaggggacaaaat

tcgttgtagatagattagaagtaactccatttgctgaccgttttccaaca

gctctaggtcgtgaaggcttgtacagaattaagggtacagatacttggat

tttggctgttgatgtaactgctgcaaagaaattaccacttcatgactacg

atctagaacataattcatacattaagttcccacaagcaacagatctctat

aatgcaagtggcagtaagataaacaccaatggtgattacattagaaaaca

atctggtcaatataaagtagacaagttattatacatttgggttccaagtg

aaaataaagcagagcctttctaccacttagtaggtaaagaagtagcatct

aataatggacatatcagctttgatgatggttatgtaaaagcaagcgatgt

tcaatttatgagcaatagtaaggcaataacaccatcaaacacagctgctg

aagcagaagctgccgcaaagaagtag

>DSM16698_SlpB

MKKNLRIVSVAAAALLAVAPVATSVVPTVGANVVQAADSNETGLGSSTGT

PTPSNSNAVTGSTPYFYMNGGEAIYNGGSFRTPFNASGFTKVSQIVSAIN

DNINFAPDGSNGNGRKEDITIAQVEKQLDALKIKYTAITDDKNGDATLDK

STLPANFDITLQHTVGNTAYRVTVPFYTTTTPTPVTGAPAFYVQKGNGAA

AETTSGQTYQVVAGSNFNPLSFTNSNGETVTFSARQSSNNGAAASISVAS

NPVDTTNAGRFYTVRLTATNNGGKTSSLSYTVLVVSNGLQKVYANGATSV

PTYSIYGNQVSSNSTTFKDGQEVYVGNTTREINKVSYTKVSTTSKANADS

SSNNLWIQTSALTQTTPSDSNTETHTVMIDSRAYDKNGNYLGHMYYAYDS

IDIVPTVVTINGKTYYKVANKDEYVRVTNITGNKRTLTHNAYIYWSSYRR

TPGTGKMYKGQTVTTYGPAMRFKNGKKYYRIEGCRNNNKRYIKAANLSAA

Q

>DSM16698_*slpB*

atgaagaagaatttaagaattgttagtgttgcagctgccgcattattggc

agttgctccggtcgcaacaagtgttgtacctactgttggtgctaacgttg

ttcaagctgctgacagcaatgaaactggtttgggtagtagtactggtaca

cctactccatcaaacagtaatgccgtaactggtagtacaccatactttta

tatgaatggtggagaagccatttataacggtggatcattcagaaccccat

ttaatgcaagtggttttacaaaggtcagccaaattgttagtgctattaat

gacaacattaactttgcacctgatggctcaaatggtaatggtcgtaagga

agacattacaattgcacaagttgagaagcaattggatgctttaaagatta

agtatacagctatcacagatgataaaaatggtgatgctactttagataag

agtaccttaccagctaactttgacattactttgcaacacactgttggtaa

tactgcttatagagtaactgttccattctatactacaactactccaactc

cagttactggtgctccagcattctacgttcaaaagggtaatggtgctgct

gctgaaactacaagtggccaaacctaccaagtagttgcaggttcaaactt

taacccattgagctttactaacagtaatggtgaaaccgtaacattctcag

cacgtcaatcaagtaacaatggcgcagctgcaagtatctcagttgcttca

aacccagtagacactactaatgcaggtcgtttctacactgttagattaac

tgctactaacaatggtggtaagactagctcactttcatacactgttttgg

tagtatcaaacggtttacaaaaggtatatgctaacggtgcaacttcagta

ccaacttacagcatctacggtaaccaagtttcatcaaactcaactacctt

taaggatggtcaagaagtttacgtaggtaacactactagagaaattaaca

aggtatcatacactaaggtttcaactacttcaaaggcaaacgctgattct

tcaagcaacaacctttggatccaaacctcagctttgacccaaactactcc

atcagacagcaacactgaaacccatactgtaatgattgactcacgtgctt

acgacaagaacggtaactacttaggccacatgtactacgcatatgacagc

attgatatcgttccaactgttgtaaccatcaacggcaagacttactacaa

ggttgctaacaaggatgaatacgttcgtgtaaccaacattactggtaaca

agcgtaccttgacccacaacgcttacatttactggtcatcataccgtcgt

accccaggtactggtaagatgtacaaaggccaaactgtaactacttacgg

tcctgcaatgagattcaagaacggtaagaagtactacagaattgaaggct

gcagaaacaacaacaagcgttacatcaaggcagctaacttgagcgcagct

caataa

>DSM16698_SlpA

MKKNLRIVSVAAAALLAVAPVAASAVSTVSADAVQSATQLGTVPALSNGD

TVNVKPNVSLNTSAYEGVKANISVSFSATVNGTTAVSNFKPGASEISLWK

EEKDKVTQVTDLQQVTSSNAGATYQVKMTNVGLNFGSQNANKKITLTFPE

GDGFKLASNNSFTNSRTIQLDKNGTVTLNEVVLHVTAKDFANPAVVNWYN

TATNSVVSTGNIELFAGSDAGKMNVAQVTSAALKKYHASNYGTAANQESS

TISYSNNLKDALKAMNVDVDAQGWFTAPKSFTFNMTATANNNDASRTLAV

TVNVPNGKDTTVPSQSKTIMHNAYYYDKDAKRVGTDKLTRYNSVTVAMNT

TTINGKAYYEVIENGKATGKFINADNIDGTKRTLKHNAYVYKSSKKRANK

VVLKKGTEVTTYGGSYTFKNGKQYYKIGNNTDKTYVKASNF

>DSM16698_*slpA*

atgaagaaaaatttaagaattgttagcgttgctgctgctgctttattagc

tgttgctccagttgctgcttctgctgtttctactgtttcagctgatgccg

ttcaatcagctactcaacttggtactgtacctgctttgtcaaatggtgac

actgttaacgttaagccaaatgtttcattaaacacttcagcttatgaagg

tgttaaggcaaacatctcagtatcattctcagctactgttaatggtacta

ctgctgtttcaaactttaagccaggtgcttcagaaatttcactttggaaa

gaagaaaaggacaaggttactcaagtaactgatttacaacaagtaacttc

atcaaacgctggtgctacttaccaagttaaaatgactaatgttggtttga

actttggctcacaaaatgccaacaagaagattactttaactttcccagaa

ggtgatggctttaagcttgcttcaaacaactcatttactaattcaagaac

cattcaacttgacaagaatggtactgtaactttaaatgaagttgtattac

acgtaactgctaaggactttgctaacccagcagttgttaactggtacaat

actgctactaactcagttgtaagtactggtaacattgaattgtttgcagg

ttcagatgctggcaagatgaacgttgctcaagttacttcagctgctttaa

agaagtaccacgcaagcaactacggtactgcagctaaccaagaatcaagc

actatttcatacagcaacaaccttaaggatgctttgaaggctatgaacgt

tgatgttgatgctcaaggttggttcactgcacctaagtcattcactttca

acatgactgctactgctaacaacaacgatgcttcacgcactttagctgta

actgttaacgtaccaaacggcaaggacactactgtaccaagccaaagcaa

gactattatgcacaacgcatactactacgacaaggacgctaagcgtgttg

gtactgacaagttgactcgttacaactcagtaactgttgctatgaacact

actactatcaacggcaaggcttactacgaagtaatcgaaaatggtaaggc

tactggtaagttcatcaacgctgacaacattgacggtactaagcgtactt

tgaagcacaacgcatacgtttacaagtcttcaaagaaacgtgctaacaag

gttgttcttaagaagggtactgaagtaactacttacggtggttcatacac

cttcaagaacggtaagcaatactacaagatcggtaacaacactgacaaga

cttacgttaaggcttcaaacttttaa

>DSM20531_SlpC

MKKNKFILTSVAALMAVSPMLSFGSQTHTVxAADTTITKTIMHTSIAYNR

DGKSTGTKYYAYKTVDVMTKPVKINDNLYYKVNGLNHYLRATNIDGVTRK

VTHNTYVYKSSNGRTSYNGRWKLYKGETVTTYGGSYKFKNGKLYFRIGGP

SKQYIKSANLGPVIKINTSVNGSSSSSTSTNLEETTVTVTTPTHLITQTS

SGYKGTAHITPVGTKFTVDRLEFNELSKRSENDDHFYHIKGTNQWINASD

VKAAKSIPLHDYFFENFSYITFPKDTDVYNADGTIQDHNGQKISKQKGxL

KVDKLVYIWVPSENKAELFYHLVGTSFYASTTPTVHWSTINVGHNAYVKA

SDVKFIDGSVKLTPSNTAAEAEAAAKK

>DSM20531_*slpC*

atgaagaaaaataaatttatcttaacttcggttgcagctttgatggctgt

aagtccaatgctttcatttggctcacaaactcatacggttgctgcagata

ctactattactaaaacgatcatgcatacttcaatagcatataacagagat

ggtaagagcacaggtaccaaatattatgcatacaagaccgttgatgtaat

gactaagcctgtaaaaataaatgacaatttgtattacaaagtaaatggat

taaatcactatttaagagctactaacattgatggtgtaacgcgtaaagta

acgcataacacttatgtttataagtcttccaatggtcgtacttcgtataa

cggcagatggaagctttataaaggtgaaacagtcactacttatggtggtt

cttacaaattcaaaaatggcaagctctacttcagaattggtggtcccagc

aaacaatacattaaatcagcaaatttaggaccagttattaaaattaatac

ttcagtgaatggatctagttcatcatcaacatctactaatttggaagaaa

caacagtcactgtaaccactccaactcatttaattactcaaaccagtagt

ggttataaaggaacggctcatattactcctgtaggaacaaaatttacagt

tgatcgtttggaatttaatgaactttctaagagaagtgaaaacgacgacc

atttttatcacatcaaaggtactaatcagtggattaatgcttctgatgtg

aaagctgctaagagtattccactacatgactatttctttgaaaacttctc

atacattacattccctaaggatactgatgtgtataatgcagacggaacta

tccaagatcataacggtcaaaaaataagtaaacaaaaaggcttgaaagta

gacaagttagtatatatttgggtaccaagtgaaaataaggcggaattatt

ctatcacttggtaggtacaagcttttatgcaagtacaacgccaaccgttc

attggagcacaattaatgttgggcataatgcttatgtaaaagctagcgat

gttaaatttattgatggaagcgtaaagctgactccatcaaacacagccgc

tgaagcagaagctgctgcgaaaaagtaa

>DSM20531_SlpB1

MKKNLRIVSVAAAALLAVAPVVSTAIPVNADTTVNVGSSTGTGANTTNTN

TQAPQNKPYFTYNNEIIGESTQSNPLGNVVRTTVSFKSGDKVSDLISKIS

KAVQFHKDNYAGGENVTINEQDFINQLKNNGVTTKTINPSKKGEKSYEEI

DKVPSTSFNITLSASANNQTATIQIPMVPEGASAPVDTTQNPQINWTKGG

QAQSASLNGQVFQVAVGSSFNPLSFINSNGDTISISAQQSKDNSTYASIE

ATSNPVNTSEAGRYYNVTLTATGNTGKKTTATYTVLITSSQKQTLYANGA

SSIPTYSIYGGNVLSNSTTFKDGDQVYVSDKTETIGNVSYSQVSTKSKSD

ANGSNMWVKTSALVKPAGDTNVKTYPVMIDSRAYDKNGNYLGHMYYAYDN

IDIVPTVVTINGKTYYKVANKDEYVRVTNITGNQRTLRHNAYIYWSSYRR

TPGTGKMYRGQTVTTYGPAMRFKNGKKYYRIEGCRNNNKRYIKAVNFY

>DSM20531_*slpB1*

atgaagaagaatttaagaattgttagtgttgcagctgctgctttgttagc

tgttgctccggtcgtttctacggctattccagttaatgctgatactactg

taaatgttggctcatcaacaggtactggtgcaaatactactaatacaaac

acccaagcacctcaaaataaaccatatttcacttataataatgaaattat

tggtgaatctactcaaagtaatcctttaggtaacgttgttcgtactactg

taagttttaagagtggtgataaagtttcagatttaattagtaaaatttct

aaagccgttcaattccacaaagataactatgctggtggcgaaaatgttac

tattaatgaacaagacttcattaatcaattgaagaataatggtgtaacca

ctaagaccattaatccttctaagaagggtgaaaaatcttacgaggaaatt

gataaggtaccaagcacttcatttaatattactttaagtgcaagtgctaa

caatcaaactgctactattcaaattcctatggtgccagaaggtgcttccg

ctccggtagataccactcaaaatccacaaattaattggactaagggcggt

caagcacaaagtgctagcttaaatggccaagtatttcaagttgctgttgg

ttcaagcttcaatccattgagcttcattaacagtaatggtgatacaattt

ctatttcagcacaacaaagtaaagataactcaacttacgcaagtattgaa

gcaacttccaacccggttaatacttcagaagcaggtcgttactacaacgt

aactttaactgcaactggtaacactggtaagaagaccactgcaacttata

ctgttttgattacttcaagtcaaaagcaaactttatatgctaatggtgca

agttcaattccaacttacagcatttacggtggtaatgttttgagtaattc

aaccacattcaaagatggcgaccaagtttacgtttcagacaagactgaaa

caattggtaatgtttcatactcacaagtttcaaccaagtctaaatcagat

gctaatggtagtaatatgtgggtaaaaacttcagcacttgtaaaaccggc

tggtgatactaacgttaagacttacccagtaatgattgactcacgtgctt

acgacaagaacggtaactatttaggccacatgtattacgcatatgacaac

attgatatcgttccaactgttgtaaccatcaacggcaagacttactacaa

ggttgctaacaaggatgaatacgttcgtgtaaccaacattactggtaacc

aacgtactttacgtcacaacgcttacatttactggtcatcataccgtcgt

accccaggtactggcaagatgtatagaggccaaactgtaactacttacgg

tcctgcaatgagattcaagaacggtaagaagtactacagaattgagggtt

gcagaaacaacaacaagcgttacattaaggctgtaaacttctattaa

>DSM20531_SlpB2

MKQVSKIMLTAVSLLSASSMLLGTPTNVKAASNVSTTSSLNSDNLYFLYN

GQKLANNAILPMEKGIAVNNGDSLEKVLNTAKSLVSLSVSGVQITTNVSE

LRGQVQSQNVLLDNSNNIAQIPTTGFYVTLTARNNGQVVNVRVPFGNAYT

VTQNAPEVQVTFNQNNVNQRLNVNNLIFQIASGSKFDPLNFGGSNKEQYK

LTATNKGTLTVDSNTVDPSQPGSWGQVKVTATNSQGQKSTSSFEVYVVPQ

GMQRLGVDIWTDSYRISDGRVWKSEQLNRGDAIYVGNDTQIINKVSYTRI

STKSQVDANSLSNNTWIKTSDLVHESVDAITKRVMHKALIYDSIGGSKLR

KISAFKLVTFEKKIYVIKNAKYYKVINAPDYIKAANVDGTKRTLKKNAYI

YATSNRRVNKNVLRKGTKITTYGSSYKFKNGKRYYRIEGATKTQKRYVRV

TNFK

>DSM20531_*slpB2*

atgaaacaagtatccaaaattatgcttactgcagttagtttattatctgc

aagcagtatgttgcttggcactcctacaaatgtcaaagcagcaagtaatg

tatcaacgactagttctttaaatagtgataatttatattttttatataat

ggtcaaaaattagctaataatgccattttacctatggaaaaaggtattgc

cgtaaataatggagatagtttagagaaagttctaaatacggctaaaagtt

tagtttctcttagtgtatctggtgttcaaattacaaccaacgtatctgaa

ttacgaggacaagtacaatcacaaaatgtgttacttgataattcaaataa

tattgctcaaataccaactactggattttatgtcacattgactgctagaa

ataatgggcaagtagtaaacgtaagagtaccattcggtaatgcatataca

gttacgcaaaatgcacccgaagttcaggtgacattcaatcaaaacaatgt

gaatcagagattgaacgtcaataatttgatctttcagattgcttcaggtt

caaaatttgatccattaaattttgggggaagtaataaagaacaatataaa

ttgacagctaccaataaaggcacattgaccgtagattctaatactgttga

tccatctcaaccaggaagttggggtcaagttaaagttaccgcaactaata

gccaaggtcagaagtctacgtctagttttgaagtttatgtagtgcctcaa

gggatgcaaagattgggcgtagatatttggactgatagttatcgcatttc

tgacggaagagtttggaagagtgagcaactgaaccgtggggatgctatat

atgttggtaatgatactcagattattaataaagtaagctacactagaata

tcgactaagagccaagtagatgccaacagtttaagtaataatacttggat

taaaactagcgatctagtacatgaaagtgtcgacgcaattacaaaacgag

tgatgcacaaagcattaatttacgatagtattggtggtagtaagctgaga

aaaatttcggctttcaaattggtgacttttgaaaagaaaatatatgtaat

taagaatgctaaatattacaaggtgatcaatgcaccagattacattaagg

ctgctaatgttgatggaactaagcgtactttaaagaaaaatgcttatatt

tacgctacaagtaatcgtcgtgtgaataagaatgttttacgcaagggtac

taaaattacaacgtatggcagttcttataagttcaagaatggtaaacgat

attatcgtattgaaggtgcaacgaaaacacaaaaacgttatgttagagta

actaattttaagtag

>DSM20531_SlpA

MKKNSRIVSFAAAALLAVAPVVATAMPVNAATTINAGSAINTNTNAKYDV

DVTPSISAVAAVAKSDTMPAISGSLTGSISASYNGKSYTANLPTDSENAT

ITDSNNTTVKPAALEAGKPYTVTVPGVSFNFGSENAGKDITIGSANPNVT

FTKDKGDQPASTVKVTLDQDGVAKLSSVQIKNVYAIDTTYNSNVNFYDVT

TGAIVTTGAVSIDADNQGQLNTASVVAAINSKYFAAQYADKKLTEDNVTF

NTETAVKDALKAQKIEVSPVGYFKAPHTFTVNVKATSKINGKSATLPVTV

TVPNVAEPVVPSQSKTVMHNAYFYDKDAKRVGTDKVTRYNTVTVAMNTTK

LANGISYYEVIENGKATGKYINADNIDGTKRTLKHNAYVYKTSKKRANKV

VLKKGTEVTTYGGSYTFKNGKQYYKIGNNTDKTYVKASNF

>DSM20531_*slpA*

atgaagaaaaattcaagaattgttagctttgctgctgctgctttattagc

tgttgcacctgttgttgcaactgctatgccagttaacgctgctactacta

ttaacgctggttcagctatcaatactaatactaatgctaagtacgatgtt

gacgtaactccaagcatatctgctgttgctgcagttgctaagagcgatac

tatgccagctatctcaggtagccttactggtagtatttcagcaagttaca

atggtaagtcatacactgctaacttaccaacggattcagaaaatgctact

attaccgatagtaataatacgactgttaagccagctgcattagaagctgg

caagccttacacagtaactgttcctggtgtttcatttaactttggttcag

aaaatgcaggtaaggatattactattggttcagctaacccaaatgtaacc

tttactaaagataaaggtgaccaacctgcttcaactgtaaaggttacgtt

agaccaagatggtgttgctaagctttcaagtgtacaaattaagaatgttt

acgcaattgacactacttacaacagcaatgtaaacttctacgatgtaaca

actggtgctattgtaacaactggtgctgtttctattgacgctgacaacca

aggtcaacttaacactgcatctgttgtagctgcaattaattctaagtact

ttgcagcacaatatgctgataagaagttgactgaggataatgttacattt

aacactgaaactgctgtcaaggatgctttaaaggctcaaaagattgaagt

aagcccagtaggttacttcaaggctccacatactttcactgttaatgtta

aggcaacttcaaaaattaacggtaagtcagctactttaccagtaactgta

actgttcctaacgttgcagaacctgttgttccaagtcaaagcaagactgt

tatgcacaacgcatacttctacgacaaggacgctaagcgtgttggtactg

acaaggtaactcgttacaacactgtaactgttgctatgaacactactaag

cttgctaacggtatttcatactacgaagtaatcgaaaacggcaaggcaac

tggcaagtacatcaacgcagacaacatcgatggtactaagcgtactttga

agcacaacgcatacgtttacaagacttcaaagaagcgtgctaacaaggtt

gttcttaagaagggtactgaagtaactacttacggtggttcatacacctt

caagaacggtaagcaatactacaagatcggtaacaacactgacaagactt

acgtaaaggcttcaaacttttaa

>GRL1112_SlpC1

MKKNKLILASVAALMAVSPVLSFGSQVHTVQAADNSVRKTVMYNSIAYDK

DGNSTGQKYYTYGSISVDPTPVTINGNQYYKISGKNQYVRVTNIDGVRRR

VTHNAYIYRTSTQKTPYGMTASSKKWKLYKGEIVTTYGGYYTFKNGKHYF

KVGGPRKQYVRTANLGPVIGTNTSTSSNNSSNTPTNNTQSVGKYETTVTV

TTPYTYLFTEVPGKIQVQRTNKRVKKGDKFVVDRLEQGTRAGTGQDGDDD

NELAIYHIKGTDYWIYNNDVQAAKQLSVQSYNKTDKSLITMDQPVEVYNA

DGTSQNIRIKKNDSAWRVDSLSYIWVAKENKAELFYRLHLNGEYRSVYRL

TNNGDYVSDRVPIKNAYIKASEVKVDPNGLKLTPSNTAAEAEAAAKK

>GRL1112_*slpC1*

atgaagaagaataaacttatcttggcttcagttgcagcattaatggctgt

aagcccagtgctttcattcggctcacaagttcacacggttcaagctgcag

ataattctgtcagaaagacagttatgtataattcaattgcttatgataaa

gatggcaattcaacaggtcaaaagtattacacttacggatcaatcagtgt

tgatccaacccctgtaactattaacggtaaccaatattacaagatttcag

gtaaaaaccaatatgttagagtaactaatattgatggtgtaagacgtaga

gtaacccacaatgcttatatttatcgtacttctactcaaaaaacgcctta

cggtatgactgcaagcagtaagaaatggaagttatacaaaggcgaaatag

taactacttatggtggctattacacctttaaaaatggtaagcactacttc

aaggtaggcggaccaagaaagcaatatgttagaactgctaacttaggtcc

agttatcggaactaatacttcaacaagttctaataattcgtcaaacaccc

caactaataatacccaatcagttggtaaatatgaaaccactgtaacagta

actactccatacacttatctttttacagaagttccaggtaaaatccaagt

ccaacgtactaataaacgtgttaaaaaaggtgataaatttgtggtagacc

gtttagaacaagggacacgtgctggtactgggcaagacggtgatgacgat

aatgagctagcaatttatcatattaagggaacggattactggatttataa

taatgatgttcaagctgctaagcaattatcagttcagagctataacaaaa

cagacaaatcattaattactatggatcaaccagttgaagtctacaatgca

gatggtacttctcaaaatattagaattaagaagaacgattcggcatggag

agttgatagcttatcatacatttgggtagccaaggaaaataaggctgaac

tattctatcgtttacatttgaatggtgaatatagaagcgtttatcgctta

acaaacaatggcgactatgtttccgatcgtgttccaattaaaaatgcata

cataaaagcaagtgaagttaaagttgatccaaatggtttgaaattaacac

catcaaacactgctgctgaagcagaggctgctgcgaaaaagtaa

>GRL1112_SlpC2

MKKNKLILASVAALMAVSPVLSFGSQTHTVQAADTTITKTIMHTSMAYDRD

GKSTGTKYYAYKTVDVMTKPVKINDNLYYKVNGLNHYLRATNIDGVTRKVT

HNTYVYKSSNGRTSYNGRWKLYKGETVTTYGGSYKFKNGKLYFRIGGPSKQ

YIKSANLGPVIKINTSVNGSSSSSTSTNLEETTVTVTTPTRLITQTSNGYK

GTAHITPVGTKFTVDRLEFNELSKRSENDDHFYHIKGTNQWINASDVKAAK

SIPLHDYFFENFSYITFPKDTDVYNADGTIQDHNGQKISKQKGQLKVDKLV

YIWVPSENKAELFYHLVGTSFYASTTPTVHWSTINVGHNAYVKASDVKFID

GSVKLTPSNTAAEAEAAAKK

>GRL1112_*slpC2*

atgaagaaaaacaaacttatcttagcttcagttgcggctttgatggctgt

aagcccggtgctttcatttggctcacagactcacacagttcaagctgcag

acactactattactaaaacaatcatgcatacttcaatggcatatgacaga

gatggtaagagcacaggtaccaaatattatgcatacaagaccgttgatgt

aatgactaagcctgtaaaaataaatgacaatttgtattacaaagtaaatg

gactaaatcactatttaagagctaccaatattgatggtgtaacgcgtaaa

gtaacgcataacacttatgtttataagtcttccaatggtcgtacttcgta

taacggcagatggaagctttataaaggtgaaacagtcactacttatggtg

gttcttacaaattcaaaaatggcaagctctacttcagaattggtggtccc

agcaaacaatacattaaatcagcaaatttaggaccagttattaaaattaa

tacttcagtgaatggatctagttcatcatcaacatctactaatttggaag

aaacaacagtcactgtaaccactccaactcgtttaattacccaaactagt

aatggctataagggaacggctcatattactccggtaggaacaaaatttac

agttgatcgtttggaatttaatgaactttctaagagaagtgaaaacgacg

accatttttatcatatcaaaggtactaatcagtggattaatgcttctgat

gtgaaagctgctaagagtattccactacatgactatttctttgaaaactt

ctcatacattacattccctaaagatactgatgtgtataatgcagacggaa

ctatccaagatcataacggtcaaaaaataagtaaacaaaaaggccaattg

aaagtagacaagttagtatatatttgggtgccaagtgaaaataaggctga

attattctatcatttggtaggtacaagcttttatgcaagtacaacgccaa

ccgttcattggagcacaattaatgttgggcataatgcttatgtaaaagcc

agcgatgttaagtttattgacggaagcgtaaagctaactccatcaaacac

agccgctgaagcagaagctgctgcgaagaagtaa

>GRL1112_SlpB

MKKNLRIISVAAAALLAVAPVATSVVPTVGANVVQAADSNETGLGSSTGT

PTPSNSNAVTGSTPYFYMNGGEAIYNGGSFRTPFNASGFTKVSQIVSAIN

DNINFAPDGSNGNGRKEDITIAQVEKQLDALKIKYTAITDDKNGDATLDK

STLPANFDITLQHTVGNTAYRVTVPFYTTATPTPVTGAPAFCVQKGNGAA

AETTSGQTYQVVAGSNFNPLSFTNSNGETVTFSARQSSNNGAAASISVAS

NPVDTTNAGRFYTVRLTATNNGGKTSSLSYTVLVVSNGLQKVYANGATSV

PTYSIYGNQVSSNSTTFKDGQEVYVGNTTREINKVSYTKVSTTSKANADT

SSNNLWIQTSALTQTTPSDSNTETHTVMIDSRAYDKNGNYLGHMYYAYDS

IDIVPTVVTINGKTYYKVANKDEYVRVTNITGNKRTLTHNAYIYWSSYRR

TPGTGKMYKGQTVTTYGPAMRFKNGKKYYRIEGCRNNNKRYIKAANLSAA

Q

>GRL1112_*slpB*

atgaagaaaaatttaagaattattagtgttgcagctgctgctttattagc

agttgctccggtcgcaactagtgttgtacctactgttggtgctaacgttg

ttcaagctgctgacagcaatgaaactggtttgggtagtagtactggtaca

cctactccatcaaacagtaatgccgtaactggtagtacaccatactttta

tatgaatggtggagaagccatttataacggtggatcattcagaaccccat

ttaatgcaagtggttttacaaaggtcagccaaattgttagtgctattaat

gacaacattaactttgcacctgatggctcaaatggtaatggtcgtaagga

agacattacaattgcacaagttgagaagcaattggatgctttaaagatta

agtatacagctatcacagatgataaaaatggtgatgctactttagataag

agtaccttaccagctaactttgacattactttgcaacacactgttggtaa

tactgcttatagagtaactgttccattctatactacagctactccaactc

cagttactggtgctccagcattctgcgttcaaaagggtaatggtgctgct

gctgaaactacaagtggccaaacctaccaagtagttgcaggttcaaactt

taacccattgagctttactaacagtaatggtgaaaccgtaacattctcag

cacgtcaatcaagtaacaatggcgcagctgcaagtatctcagttgcttca

aacccagtagacactactaatgcaggtcgtttctacactgttagattaac

tgctactaacaatggtggtaagactagctcactttcatacactgttttgg

tagtatcaaacggtttacaaaaggtatatgctaacggtgcaacttcagta

ccaacttacagcatctacggtaaccaagtttcatcaaactcaactacctt

taaggatggtcaagaagtttacgtaggtaacactactagagaaattaaca

aggtatcatacactaaggtttcaactacttcaaaggcaaacgctgatact

tcaagcaacaacctttggatccaaacctcagctttgacccaaactactcc

atcagacagcaacactgaaacccatactgtaatgattgactcacgtgctt

acgacaagaacggtaactacttaggccacatgtactacgcatatgacagc

attgatatcgttccaactgttgtaaccatcaacggcaagacttactacaa

ggttgctaacaaggatgaatacgttcgtgtaaccaacattactggtaaca

agcgtaccttgacccacaacgcttacatttactggtcatcataccgtcgt

accccaggtactggtaagatgtacaaaggccaaactgtaactacttacgg

tcctgcaatgagattcaagaacggtaagaagtactacagaattgaaggct

gcagaaacaacaacaagcgttacatcaaggcagctaacttgagcgcagct

caataa

>GRL1112_SlpA

MKKNLRIVSAAAAALLAVAPVAASAVSTVSADVNTNIVLGGTTAPAVKGD

VNVTSNVQAITSPQTTTIDNQTGAVTYSNWDGKVNGTVTATYNGQSYTAT

LNETAGKENSRVTPWYTQDGGKTWNVLKKDGGVYRLEPAGKYQLSVNNVS

FNFGTANANKKNITLTSSNGVQFRENGQWKDSIKVSTDQNGAVSQPLTLL

IPITPVDVTNAKSVSFYEIANGNEVHTGSLNMTANPTSHELNVSAVLAAA

KAKYAAHQLENGASNGASVAVTTDVKDLTDQLTKAGIKVDPLGNFQAQAS

FSFNLAAKSAQNAATATLPITVSVANAAQTPAAQETTKNVTIMHISTIYD

KTGKATNEPALRAYDTVSVVSDPVTINNAKFYKLAGKDQYIKVGNVDGTS

RTLKHNSYVYKSSGKRANKKTLKKGSSVTTYGKSFMIAGHQMYRIGKNQY

VKKANF

>GRL1112_*slpA*

atgaagaaaaatttaagaattgttagcgctgctgctgctgctttattagc

tgttgctcctgttgctgctagcgccgtttctactgttagtgccgacgtta

acactaacattgttttaggtggaaccactgcaccagctgttaaaggcgat

gttaatgtaacttcaaacgttcaagccattacttcaccacaaaccactac

tattgataatcaaactggtgctgttacatacagcaactgggatggtaagg

taaacggcactgttacagctacctacaatggtcaaagttatactgcaact

cttaatgaaactgcaggtaaggaaaacagcagagttaccccttggtacac

ccaagatggtggcaaaacttggaatgttcttaaaaaagacggtggcgttt

accgtctagaaccagctggtaagtaccaacttagtgttaacaatgtatca

tttaactttggtactgctaacgcaaacaagaagaacattactttgactag

ctcaaatggtgtacaattccgtgaaaacggtcaatggaaggactcaatta

aagtttcaaccgaccaaaacggtgctgtttcacaacctttaactttatta

attccaattacaccagttgatgtaactaatgcaaagagcgtatcattcta

cgaaattgcaaatggtaatgaagtacatactggtagccttaacatgactg

ctaacccaacttcacacgaacttaacgttagtgctgttcttgcagctgct

aaggctaagtatgctgctcaccaacttgaaaatggtgcaagcaacggtgc

ttcagttgcagttactactgatgtaaaggaccttactgatcaattaacta

aggccggtatcaaggtggatccattgggcaacttccaagctcaagcttca

ttcagcttcaacttggctgctaagtcagcacaaaacgctgcaactgcaac

cttaccaattactgtttcagttgcaaacgctgctcaaactccagctgctc

aagaaactactaagaacgtaactattatgcacatttcaactatttacgac

aagactggtaaggctactaacgaaccagcattgcgtgcatacgatactgt

atcagtagtttcagaccctgtaactatcaacaatgctaagttctacaagc

ttgctggtaaggaccaatacatcaaggttggtaacgttgacggtacttca

agaactttgaagcacaactcatacgtttacaagtcatctggtaagcgtgc

aaacaagaagactttgaagaagggttcatcagtaactacttacggtaagt

cattcatgattgctggtcaccaaatgtacagaattggcaagaaccaatac

gtaaagaaggcaaacttctaa

>GRL1114_SlpC1

MKKNKLILASVAALMAVSPVLSFGSQVHTVQAADNSVRKTVMYNSIAYDK

DGNSTGQKYYTYGSISVDPTPVTINGNQYYKISGKNQYVRVTNIDGVRRR

VTHNAYIYRTSTQKTPYGMTASSKKWKLYKGEIVTTYGGYYTFKNGKHYF

KVGGPRKQYVRTANLGPVIGTNTSTSSNNSSNTPTNNTQSVGKYETNVTV

TTPYTYLFTEVPGKIQVQRTNKRVKKGDKFVVDRLEQGTRAGTGQDGDDD

NELAIYHIKGTDYWIYNNDVQAAKQLSVQSYNKTDKSLITMDQPVEVYNA

DGTSQNIRIKKNDSAWRVDSLSYIWVAKENKAELFYRLHLNGEYRSVYRL

TNNGDYVSDRVPIKNAYIKASEVKVDPNGLKLTPSNTAAEAEAAAKK

>GRL1114_*slpC1*

atgaagaagaataaacttatcttggcttcagttgcagcattaatggctgt

aagcccagtgctttcattcggctcacaagttcacacggttcaagctgcag

ataattctgtcagaaagacagttatgtataattcaattgcttatgataaa

gatggcaattcaacaggtcaaaagtattacacttacggatcaatcagtgt

tgatccaacccctgtaactattaacggtaaccaatattacaagatttcag

gtaaaaaccaatatgttagagtaactaatattgatggtgtaagacgtaga

gtaacccacaatgcttatatttatcgtacttctactcaaaaaacgcctta

cggtatgactgcaagcagtaagaaatggaagttatacaaaggcgaaatag

taactacttatggtggctattacacctttaaaaatggtaagcactacttc

aaggtaggcggaccaagaaagcaatatgttagaactgctaacttaggtcc

agttatcggaactaatacttcaacaagttctaataattcgtcaaacaccc

caactaataatacccaatcagttggtaaatatgaaaccaatgtaacagta

actactccatacacttatctttttacagaagttccaggtaaaatccaagt

ccaacgtactaataaacgtgttaaaaaaggtgataaatttgtggtagacc

gtttagaacaagggacacgtgctggtactgggcaagacggtgatgacgat

aatgagctagcaatttatcatattaagggaacggattactggatttataa

taatgatgttcaagctgctaagcaattatcagttcagagctataacaaaa

cagacaaatcattaattactatggatcaaccagttgaagtctacaatgca

gatggtacttctcaaaatattagaattaagaagaacgattcggcatggag

agttgatagcttatcatacatttgggtagccaaggaaaataaggctgaac

tattctatcgtttacatttgaatggtgaatatagaagcgtttatcgctta

acaaacaatggcgactatgtttccgatcgtgttccaattaaaaatgcata

cataaaagcaagtgaagttaaagttgatccaaatggtttgaaattaacac

catcaaacactgctgctgaagcagaggctgctgcgaaaaagtaa

>GRL1114_SlpC2

MIKMKKNKLILALVAALMAVSPVLSFGSQTHTVQAADTTITKTIMHTSMA

YDRDGKSTGTKCYAYKTVDVVTKPVKINGNLYYKVNGLNHYLRATNIDGV

TRKVTHNTYVYKSSNGRTSYNGRWKLYKGETVTTYGGSYKFKNGKHYFRI

GGPSKQYIKSANLGPVIKTNTSVNGSGSSSASTNSEETTVTVTTPTRLIT

QTSNGYKGTAHITPVGTKFTVDRLEFNELSKRSENDDHFYHIKGTDQWIN

ASDVKAAKSIPLHDYFFENFSYITFPKDTDVYNADGTIQDHNGQKISKQK

GQLKVDKLVYIWVPSENKAELFYHLVGTSFYASTTPTVHWSTINVGHNAY

VKASDVKFIDGSVKLTPSNTAAEAEAAAKK

>GRL1114_*slpC2*

atgattaaaatgaagaaaaacaaacttatcttagctttagttgcggcttt

gatggctgtaagcccggtgctttcatttggctcacagactcacacagttc

aagctgcagacactactattactaaaacaatcatgcatacttcaatggca

tatgacagagatggtaagagcacaggtaccaaatgttatgcatacaagac

cgttgatgtagtgactaagcctgtaaaaataaacggtaatttgtattaca

aagtaaatggattaaatcactatttaagagctaccaatattgatggtgta

acgcgtaaagtaacgcataacacttatgtttataagtcttccaatggtcg

tacttcgtataacggcagatggaagctttataaaggtgaaacagtcacta

cttatggtggttcttacaaattcaaaaatggtaagcactacttcagaatt

ggtggtccaagcaaacaatacattaaatcagccaatttaggaccagttat

taaaactaatacttcagtgaatggatctggttcatcatcagcatctacta

attcggaagaaacaacagtcactgtaaccactccgactcgtttaattacc

caaaccagtaatggctataaaggaacggctcatattactcctgtaggaac

aaaatttacagttgatcgtttggaatttaatgaactttctaagagaagtg

aaaacgacgaccatttttatcacatcaaaggtactgatcagtggatcaat

gcttctgatgtgaaagctgctaaaagtattccactacatgactatttctt

tgaaaacttctcatacattacattccctaaggacactgatgtgtataatg

cagacggaactatccaagatcataacggccaaaaaataagtaaacaaaaa

ggtcaattaaaagtagacaagttagtatatatttgggtaccaagtgaaaa

taaggctgaattattctatcacttggtaggcacaagtttttatgcaagta

caacgccaaccgttcattggagcacaattaatgttggtcataatgcttat

gtaaaagctagcgatgttaaatttattgatggaagcgtaaaattaactcc

atcaaacacagccgctgaagcagaagctgctgcaaagaagtaa

>GRL1114_SlpB

MKKNLRIISVAAAALLAVAPVVSTAIPVNADTAVNVGSSTGTGANTTNTN

TQAPQNKPYFTYNNEIIGESTQSNPLGNVVRTTVSFKSGDKVSDLISKIS

KAVQFHKDNYAGGENVTINEQDFINQLKNNGVTTKTINPSKKGEKSYEEI

DKVPSTSFNITLSASANNQTATIQIPMVPEGASAPVDTTQNPQINWTKGG

QAQSASLNGQVFQVAVGSSFNPLSFTNSNGDTISISAQQSKDNSTYASIE

ATSNPVNTSEAGRYYNVTLTATGNTGKKTTATYTVLITSSQKQTLYANGA

SSIPTYSIYGGNVLSNSTTFKDGDQVYVSDKTETIGNVSYSQVSTKSKSD

ANGSNMWVKTSALVKPAGDTNVKTYPVMIDSRAYDKNGNYLGHMYYAYDN

IDVVPTVVTINGKTYYKVANKDEYVRVTNITGNKRTLTHNAYIYWSSYRR

TPGTGKMYKGQTVTTYGPAMRFKNGKKYYRIEGCRNNNKRYIKAANLSAA

Q

>GRL1114_*slpB*

atgaagaagaatttaagaattattagtgttgcagctgctgctttgttagc

tgttgctccggtcgtttctacggctattccagttaatgctgatactgctg

taaatgttggctcatcaacaggtactggtgcaaatactactaatacaaac

acccaagcacctcaaaataaaccatatttcacttataataatgaaattat

tggtgaatctactcaaagtaatcctttaggtaacgttgttcgtactactg

taagttttaagagtggtgataaagtttcagatttaattagtaaaatttct

aaagccgttcaattccacaaagataactatgctggtggcgaaaatgttac

tattaatgaacaagacttcattaatcaattgaagaataatggtgtaacca

ctaagaccattaatccttctaagaagggtgaaaaatcttacgaggaaatt

gataaggtaccaagcacttcatttaatattactttaagtgcaagtgctaa

caatcaaactgctactattcaaattcctatggtgccagaaggtgcttccg

ctccggtagataccactcaaaatccacaaattaattggactaagggcggt

caagcacaaagtgctagcttaaatggccaagtatttcaagttgctgttgg

ttcaagcttcaatccattgagcttcactaacagtaatggtgatacaattt

ctatttcagcacaacaaagtaaagataactcaacttacgcaagtattgaa

gcaacttccaacccggttaatacttcagaagcaggtcgttactacaacgt

aactttaactgcaactggtaacactggtaagaagaccactgcaacttata

ctgttttgattacttcaagtcaaaagcaaactttatatgctaatggtgca

agttcaattccaacttacagcatttacggtggtaatgttttgagtaattc

aaccacattcaaagatggcgaccaagtttacgtttcagacaagactgaaa

caattggtaatgtttcatactcacaagtttcaaccaagtctaaatcagat

gctaatggtagtaatatgtgggtaaaaacttcagcacttgtaaaaccggc

tggtgatactaacgttaagacttacccagtaatgattgactcacgtgctt

acgacaagaacggtaactatttaggccacatgtattacgcatatgacaac

attgatgtcgttccaactgttgtaaccatcaacggcaagacttactacaa

ggttgctaacaaggatgaatacgttcgtgtaaccaacattactggtaaca

agcgtaccttgacccacaacgcttacatttactggtcatcataccgtcgt

accccaggtactggtaagatgtacaaaggccaaactgtaactacttacgg

tcctgcaatgagattcaagaacggtaagaagtactacagaattgaaggct

gcagaaacaacaacaagcgttacatcaaggcagctaacttgagcgcagct

caataa

>GRL1114_SlpA

MKKNLRIVSVAAAALLAVAPVAATAMPVNAATTVNINGNTSTPVAQNADV

NLATNFTAIAYVAGQNGAQGTNGVVSGSVTATYNGQSYTGNLTDGNAKDT

TIYSVSDKKPVDVSSSAFAAGQYYAVIKDVSFNFGSQNAGKKLTVSLKGG

LLTTTDANAKAAESVTVTLDKNGVANFAEVQTPNFKAVNPFSTSTVAWYN

NNAVATSANVTVNAGNNNLVNVSQIVAALNGYTAHELTRGDNGQVASNPV

TSPITAAAVTDQLKAQNIAVDGAGYFTAPTSLSLKFTATANSSNASAELP

VTVSIPNGKVTTVESVSKTVMHNAYYYDKDAKRVGTDKLTRYNSVTVSPK

TTTIKGKAYYEVVENGKLSGKFINADNIDGTKRTLKHNAYVYASSKKRAN

KVVLKKGTEVTTYGGSYTFKNGKQYYKIGNNTDKTYVKASNF

>GRL1114_*slpA*

atgaagaaaaatttaagaattgttagcgttgctgctgctgctttattagc

tgttgctccagttgctgcaactgctatgccagttaacgctgctactacag

ttaatatcaacggtaacacttcaacaccagttgcacaaaatgcagatgta

aaccttgctactaactttactgcaatcgcttatgttgcaggtcaaaacgg

tgctcaaggtacaaacggtgttgtttcaggttctgtaactgcaacttaca

atggtcaaagctacactggtaacttaactgatggtaatgctaaggatact

actatctacagtgtttcagataaaaaacctgttgatgttagttcatcagc

atttgctgcaggtcaatactacgctgtaattaaagacgtttcattcaact

ttggttcacaaaacgcaggtaagaagctcactgttagtcttaagggtggt

ttattaactactactgacgcaaatgctaaggctgctgaaagcgttactgt

aactttggataagaatggtgttgcaaactttgcagaagttcaaactccaa

actttaaggctgtaaacccattcagcacttcaactgttgcttggtacaac

aacaatgctgtcgcaacttcagctaacgtaactgttaacgcaggtaacaa

taacttggttaatgttagccaaatcgttgcagctttaaacggctacactg

cacacgaattaactcgtggtgacaatggtcaagtagcttcaaaccctgta

acttcacctattactgctgctgcagttactgatcaattaaaggcacaaaa

tattgctgttgatggtgctggttactttactgcaccaacttcattaagct

tgaaatttactgctactgcaaacagtagtaatgcttctgctgaattacca

gttactgtttcaattccaaacggtaaggtaactactgttgaaagcgtatc

aaagactgttatgcacaacgcatactactacgacaaggatgctaagcgtg

ttggtactgacaagttgactcgttacaactcagtaactgtttcacctaag

actactaccatcaaaggcaaggcttactacgaagtagttgaaaacggcaa

gctttcaggcaagttcatcaacgcagacaacatcgatggtactaagcgta

ctttgaagcacaacgcttacgtttacgcatcatcaaagaagcgtgctaac

aaggttgttcttaagaagggtactgaagtaactacttacggtggttcata

caccttcaagaacggtaagcaatactacaagatcggtaacaacactgaca

agacttacgttaaggcttcaaacttttaa

>GRL1115_SlpC

MKKNKLILASVAALMAVSPVLSFGSQVHTVQAADNSVRKTVMHNSIAYDK

DGNSTGQKYYTYGSISVDPTPVTINGNQYYKISGKNQYVRVTNIDGVRRR

VTHNAYIYRTSTQKTPYGMTASSKKWKLYKGEIVTTYGGYYTFKNGKHYF

KVGGPRKQYVRTANLGPVIGTNTSTSSNNSSNTPTNNTQSVGKYETTVTV

TTPYTYLFTEVPGKIQVQRTNKRVKKGDKFVVDRLEQGTRAGTGQDGDDD

NELAIYHIKGTDYWIYNNDVQAAKQLSVQSYNKTDKSLITMDQPVEVYNA

DGTSQNIRIKKNDSAWRVDSLSYIWVAKENKAELFYRLHLNGEYRSVYRL

TNNGDYVSDRVPIKNAYIKASEVKVDPNGLKLTPSNTAAEAEAAAKK

>GRL1115_*slpC*

atgaagaagaataaacttatcttggcttcagttgcagcattaatggctgt

aagcccagtgctttcattcggctcacaagttcacacggttcaagctgcag

ataattctgtcagaaagacagttatgcataattcaattgcttatgataaa

gatggcaattcaacaggtcaaaagtattacacttacggatcaatcagtgt

tgatccaacccctgtaactattaacggtaaccaatattacaagatttcag

gtaaaaaccaatatgttagagtaactaatattgatggtgtaagacgtaga

gtaacccacaatgcttatatttatcgtacttctactcaaaaaacgcctta

cggtatgactgcaagcagtaagaaatggaagttatacaaaggcgaaatag

taactacttatggtggctattacacctttaaaaatggtaagcactacttc

aaggtaggcggaccaagaaagcaatatgttagaactgctaacttaggtcc

agttatcggaactaatacttcaacaagttctaataattcgtcaaacaccc

caactaataatacccaatcagttggtaaatatgaaaccactgtaacagta

actactccatacacttatctttttacagaagttccaggtaaaatccaagt

ccaacgtactaataaacgtgttaaaaaaggtgataaatttgtggtagacc

gtttagaacaagggacacgtgctggtactgggcaagacggtgatgacgat

aatgagctagcaatttatcatattaagggaacggattactggatttataa

taatgatgttcaagctgctaagcaattatcagttcagagctataacaaaa

cagacaaatcattaattactatggatcaaccagttgaagtctacaatgca

gatggtacttctcaaaatattagaattaagaagaacgattcggcatggag

agttgatagcttatcatacatttgggtagccaaggaaaataaggctgaac

tattctatcgtttacatttgaatggtgaatatagaagcgtttatcgctta

acaaacaatggcgactatgtttccgatcgtgttccaattaaaaatgcata

cataaaagcaagtgaagttaaagttgatccaaatggtttgaaattaacac

catcaaacactgctgctgaagcagaggctgctgcgaaaaagtaa

>GRL1115_SlpB

MKKNLRIVSVAAAALLAVAPVVSTAIPVNADTAVNVGSSTGTGANTTNTN

TQAPQNKPYFTYNNEIIGESTQSNPLGNVVRTTVSFKSGDKVSDLISKIS

KAVQFHKDNYAGGENVTINEQDFINQLKNNGVTTKTINPSKKGEKSYEEI

DKVPSTSFNITLSASANNQTATIQIPMVPEGASAPVDTTQNPQINWTKGG

QAQSASLNGQVFQVAVGSSFNPLSFTNSNGDTISISAQQSKDNSTYASIE

ATSNPVNTSEAGRYYNVTLTATGNTGKKTTATYTVLITSSQKQTLYANGA

SSIPTYSIYGGNVLSNSTTFKDGDQVYVSDKTETIGNVSYSQVSTKSKSD

ANGSNMWVKTSALVKPAGDTNVKTYPVMIDSRAYDKNGNYLGHMYYAYDN

IDIVPTVVTINGKTYYKVANKDEYVRVTNITGNKRTLTHNAYIYWSSYRR

TPGTGKMYKGQTVTTYGPAMRFKNGKKYYRIEGCRNNNKRYIKAANLSAA

Q

>GRL1115_*slpB*

atgaagaagaatttaagaattgttagtgttgcagctgctgctttgttagc

tgttgctccggtcgtttctacggctattccagttaatgctgatactgctg

taaatgttggctcatcaacaggtactggtgcaaatactactaatacaaac

acccaagcacctcaaaataaaccatatttcacttataataatgaaattat

tggtgaatctactcaaagtaatcctttaggtaacgttgttcgtactactg

taagttttaagagtggtgataaagtttcagatttaattagtaaaatttct

aaagccgttcaattccacaaagataactatgctggtggcgaaaatgttac

tattaatgaacaagacttcattaatcaattgaagaataatggtgtaacca

ctaagaccattaatccttctaagaagggtgaaaaatcttacgaggaaatt

gataaggtaccaagcacttcatttaatattactttaagtgcaagtgctaa

caatcaaactgctactattcaaattcctatggtgccagaaggtgcttccg

ctccggtagataccactcaaaatccacaaattaattggactaagggcggt

caagcacaaagtgctagcttaaatggccaagtatttcaagttgctgttgg

ttcaagcttcaatccattgagcttcactaacagtaatggtgatacaattt

ctatttcagcacaacaaagtaaagataactcaacttacgcaagtattgaa

gcaacttccaacccggttaatacttcagaagcaggtcgttactacaacgt

aactttaactgcaactggtaacactggtaagaagaccactgcaacttata

ctgttttgattacttcaagtcaaaagcaaactttatatgctaatggtgca

agttcaattccaacttacagcatttacggtggtaatgttttgagtaattc

aaccacattcaaagatggcgaccaagtttacgtttcagacaagactgaaa

caattggtaatgtttcatactcacaagtttcaaccaagtctaaatcagat

gctaatggtagtaatatgtgggtaaaaacttcagcacttgtaaaaccggc

tggtgatactaacgttaagacttacccagtaatgattgactcacgtgctt

acgacaagaacggtaactatttaggccacatgtattacgcatatgacaac

attgatatcgttccaactgttgtaaccatcaacggcaagacttactacaa

ggttgctaacaaggatgaatacgttcgtgtaaccaacattactggtaaca

agcgtaccttgacccacaacgcttacatttactggtcatcataccgtcgt

accccaggtactggtaagatgtacaaaggccaaactgtaactacttacgg

tcctgcaatgagattcaagaacggtaagaagtactacagaattgaaggct

gcagaaacaacaacaagcgttacatcaaggcagctaacttgagcgcagct

caataa

>GRL1115_SlpA

MKKNLRIVSVAAAALLAVAPVAASAVSTVSAADATTTTTATTTNKPTIDL

TGAGAVTNAADTVTVTPNFTLTAAVAKDKKVTASATLQGTITASLNGTSV

TANVIDAAKDVTLKSNTGYTTIYKYDSTTKTTENNLGKWNEATNDVYVKA

GNDYQVELTGVGFSFGSANANKKLSLKLPSNVTVTGAAVNGNEVTLDQYG

NVTNLTFTVKNIKAYDATNTSAVQFYNTNSGLIESKAASYMDLANNNGNL

NVNTLLDGLKKQYKAVQLQNGELKDVNVTTTAADLTAELEKAGIKVNAAG

DFEAPASFTATLTAKSTVNGKVATLPVTVTVPNGKVTTVPSQTKTIMHNA

YYYDKDAKRVGTDKVTRYNTVTVAMNTTKLANGISYYEVIENGKATGKFI

NADNIDGTKRTLKHNAYVYKSSKKRANKVVLKKGTEVTTYGGSYTFKNGK

QYYKIGNNTDKTYVKVANF

>GRL1115_*slpA*

atgaagaaaaatttaagaattgttagcgttgctgctgctgctttattagc

tgttgctccagttgctgcttctgctgtatctactgtttcagctgctgacg

ctactacaactactactgctactacaaccaacaagccaactattgactta

actggtgcaggtgctgttactaacgcagctgatactgttactgtaactcc

taactttactttgactgctgctgttgctaaggataagaaagttactgctt

cagcaactttacaaggtactattactgcttcacttaacggtacttcagta

actgctaacgtaattgacgctgctaaggatgttactttaaagagtaacac

aggctacactactatttacaagtacgattctacaactaagaccactgaaa

acaaccttggtaagtggaacgaagcaactaacgatgtttacgtaaaagct

ggtaacgactaccaagttgaacttactggtgtaggcttcagctttggctc

agctaacgcaaacaagaaattatcacttaagttgccttcaaacgtaactg

ttacaggtgctgcagttaatggcaacgaagtaactttagaccaatacggt

aacgttactaacttaacctttactgtaaagaacattaaggcttacgatgc

tactaacactagtgctgttcaattctacaacactaactcaggcttaattg

aaagcaaggctgcatcatacatggatcttgctaacaacaacggtaacctt

aatgtaaacactcttcttgacggtttgaagaagcaatacaaagctgttca

acttcaaaatggtgaacttaaggacgttaatgtaaccactactgctgcag

accttactgctgaacttgaaaaggctggtattaaggtaaatgctgctggt

gactttgaagctcctgcatcattcactgcaactttaactgctaagtcaac

agttaacggtaaggttgctactttgcctgtaactgtaactgttccaaacg

gcaaggtaactactgtaccaagtcaaactaagactattatgcacaacgca

tactactacgacaaggatgctaagcgtgttggtactgacaaggtaactcg

ttacaacactgtaactgttgctatgaacactactaagcttgctaacggta

tttcatactacgaagtaatcgaaaacggtaaggcaactggcaagttcatc

aacgctgacaacatcgatggtactaagcgtactttgaagcacaacgcata

cgtttacaagtcatcaaagaagcgtgctaacaaggttgttcttaagaagg

gtactgaagtaactacttacggtggttcatacaccttcaagaacggtaag

caatactacaagatcggtaacaacactgacaagacttacgttaaggttgc

aaacttttaa

>GRL1116_SlpC1

MKKNKLILASVAALMAVSPVLSFGSQVHTVQAADNSVRKTVMYNSIAYDK

DGNSTGQKYYTYGSISVDPTPVTINGNQYYKISGKNQYVRVTNIDGVRRR

VTHNAYIYRTSTQKTPYGMTASSKKWKLYKGEIVTTYGGYYTFKNGKHYF

KVGGPRKQYVRTANLGPVIGTNTSTSSNNSSNTTTNNTQPVGKDETTVTV

TTPYTYLFTEVPGKIEVQRTNKRVKKGDKFVVDRLEQGTRAGTGQDGDDD

NELAIYHIKGTDYWIYNNDVQAAKQLSVQSYNKTDKSLITMDQPVEVYNA

DGTSQNIRIKKNDLVWRVDSLSYIWVAKENKAELFYRLHLNGEYRSVYRL

TNNGDYVSDRVPIKNAYIKASEVKVDPNGLKLTPSNTAAEAEAAAKK

>GRL1116_*slpC1*

atgaagaagaataaacttatcttggcttcagttgcagcattaatggctgt

aagcccagtgctttcattcggctcacaagttcacacggttcaagctgcag

ataattctgtcagaaagacagttatgtataattcaattgcttatgataaa

gatggcaattcaacaggtcaaaagtattacacttacggatcaatcagtgt

tgatccaacccctgtaactattaacggtaaccaatattacaagatttcag

gtaaaaaccaatatgttagagtaactaatattgatggtgtaagacgtaga

gtaacccacaatgcttatatttatcgtacttctactcaaaaaacacctta

cggtatgactgcaagcagtaagaaatggaagttatacaaaggcgaaatag

taactacttatggtggctactacacttttaagaatggcaagcactacttc

aaggtaggcggaccaagaaagcaatatgtaagaactgctaacttaggtcc

agttatcggaactaatacttcaacaagttctaataattcgtcaaacacca

caaccaataatacccaaccagttggtaaagatgaaaccactgtaacggta

actactccatatacttatctatttacagaagtgcctggcaagattgaagt

tcaacgtactaataaacgtgttaaaaaaggtgataagtttgtagtagacc

gtttagaacaagggacacgtgctggtactgggcaagacggtgatgacgat

aatgagctagcaatttatcatattaagggaacagattattggatttataa

taatgatgttcaagctgctaagcaattatcagttcagagttataacaaaa

cagacaaatcattaattactatggatcaaccagttgaagtctacaatgca

gatggtacttctcaaaatattagaattaagaagaacgatttggtatggag

agttgatagcttatcatacatttgggtagccaaggaaaataaggctgaac

tattctatcgtttacatttgaatggtgaatatagaagcgtttatcgctta

acaaacaatggcgactatgtttccgatcgtgttccaattaaaaatgcata

cataaaagcaagtgaagttaaagttgatccaaatggtttgaaattaacac

catcaaacactgccgctgaagcagaggctgctgcgaaaaagtaa

>GRL1116_SlpC2

MKKNKLILASVAALMAVSPVLSFGSQTHTVQAADTTITKTIMHTSMAYDR

DGKSTGTKYYAYKTVDVMTKPVKINGNLYYKVNGLNHYLRATNIDGVTRK

ITHNTYIYKSSNGRTSFNGRWKLYKGETVTTYGGSYKFKNGKHYFRIGGP

SKQYIKSANLGPVIKTNTSVNGSGSSSASTNSEETTVTVTTPTRLITQTS

NGYKGTAHITPVGTKFTVDRLEFNELSKRSENDDHFYHIKGTDQWINASD

VKAAKSIPLHDYFFENFSYITFPKDTDVYNADGTIQDHNGQKISKQKGQL

KVDKLVYIWVPSENKAELFYHLVGTSFYASTTPTVHWSTINVGHNAYVKA

SDVKFIDGSVKLTPSNTAAEAEAAAKK

>GRL1116_*slpC2*

atgaagaaaaacaaacttatcttagcttcagttgcggctttgatggctgt

aagcccggtgctttcatttggctcacagactcacacagttcaagctgcag

acactactattactaaaacaatcatgcatacttcaatggcatatgacaga

gatggtaagagcacaggtaccaaatattatgcatacaagaccgttgatgt

gatgactaagcctgtaaaaataaatggcaatttgtattacaaagtaaatg

gattaaatcactatttaagagctaccaatattgatggtgtaacgcgtaaa

ataacgcataatacctatatttataaatcttctaatggtcgaacttcatt

taatggtagatggaagctttataaaggtgaaacagtcaccacttatggcg

gttcttacaaattcaaaaatggcaagcactacttcagaattggtggtcca

agcaaacaatacattaaatcagccaatttaggaccagttattaaaactaa

tacttcagtgaatggatctggttcatcatcagcatctactaattcggaag

aaacaacagtcactgtaaccactccgactcgtttaattacccaaaccagt

aatggctataaaggaacggctcatattactcctgtaggaacaaaatttac

agttgatcgtttggaatttaatgaactttctaagagaagtgaaaacgacg

accatttttatcacatcaaaggtactgatcagtggatcaatgcttctgat

gtgaaagctgctaaaagtattccactacatgactatttctttgaaaactt

ctcatacattacattccctaaggacactgatgtgtataatgcagacggaa

ctatccaagatcataacggccaaaaaataagtaaacaaaaaggtcaatta

aaagtagacaagttagtatatatttgggtaccaagtgaaaataaggctga

attattctatcacttggtaggcacaagtttttatgcaagtacaacgccaa

ccgttcattggagcacaattaatgttggtcataatgcttatgtaaaagct

agcgatgttaaatttattgatggaagcgtaaaattaactccatcaaacac

agccgctgaagcagaagctgctgcaaagaagtaa

>GRL1116_SlpB

MKKNLRIISVAAAALLAVAPVVSTAIPVNADTAVNVGSSTGTGANTTNTN

TQAPQNKPYFTYNNEIIGESTQSNPLGNVVRTTVSFKSGDKVSDLISKIS

KAVQFHKDNYAGGENVTINEQDFINQLKNNGVTTKTINPSKKGEKSYEEI

DKVPSTSFNITLSASANNQTATIQIPMVPEGASAPVDTTQNPQINWTKGG

QAQSASLNGQVFQVAVGSSFNPLSFTNSNGDTISISAQQSKDNSTYASIE

ATSNPVNTSEAGRYYNVTLTATGNTGKKTTATYTVLITSSQKQTLYANGA

SSIPTYSIYGGNVLSNSTTFKDGDQVYVSDKTETIGNVSYSQVSTKSKSD

ANGSNMWVKTSALVKPAGDTNVKTYPVMIDSRAYDKNGNYLGHMYYAYDN

IDVVPTVVTINGKTYYKVANKDEYVRVTNITGNKRTLTHNAYIYWSSYRR

TPGTGKMYKGQTVTTYGPAMRFKNGKKYYRIEGCRNNNKRYIKAANLSAA

Q

>GRL1116_*slpB*

atgaagaagaatttaagaattattagtgttgcagctgctgctttgttagc

tgttgctccggtcgtttctacggctattccagttaatgctgatactgctg

taaatgttggctcatcaacaggtactggtgcaaatactactaatacaaac

acccaagcacctcaaaataaaccatatttcacttataataatgaaattat

tggtgaatctactcaaagtaatcctttaggtaacgttgttcgtactactg

taagttttaagagtggtgataaagtttcagatttaattagtaaaatttct

aaagccgttcaattccacaaagataactatgctggtggcgaaaatgttac

tattaatgaacaagacttcattaatcaattgaagaataatggtgtaacca

ctaagaccattaatccttctaagaagggtgaaaaatcttacgaggaaatt

gataaggtaccaagcacttcatttaatattactttaagtgcaagtgctaa

caatcaaactgctactattcaaattcctatggtgccagaaggtgcttccg

ctccggtagataccactcaaaatccacaaattaattggactaagggcggt

caagcacaaagtgctagcttaaatggccaagtatttcaagttgctgttgg

ttcaagcttcaatccattgagcttcactaacagtaatggtgatacaattt

ctatttcagcacaacaaagtaaagataactcaacttacgcaagtattgaa

gcaacttccaacccggttaatacttcagaagcaggtcgttactacaacgt

aactttaactgcaactggtaacactggtaagaagaccactgcaacttata

ctgttttgattacttcaagtcaaaagcaaactttatatgctaatggtgca

agttcaattccaacttacagcatttacggtggtaatgttttgagtaattc

aaccacattcaaagatggcgaccaagtttacgtttcagacaagactgaaa

caattggtaatgtttcatactcacaagtttcaaccaagtctaaatcagat

gctaatggtagtaatatgtgggtaaaaacttcagcacttgtaaaaccggc

tggtgatactaacgttaagacttacccagtaatgattgactcacgtgctt

acgacaagaacggtaactatttaggccacatgtattacgcatatgacaac

attgatgtcgttccaactgttgtaaccatcaacggcaagacttactacaa

ggttgctaacaaggatgaatacgttcgtgtaaccaacattactggtaaca

agcgtaccttgacccacaacgcttacatttactggtcatcataccgtcgt

accccaggtactggtaagatgtacaaaggccaaactgtaactacttacgg

tcctgcaatgagattcaagaacggtaagaagtactacagaattgaaggct

gcagaaacaacaacaagcgttacatcaaggcagctaacttgagcgcagct

caataa

>GRL1116_SlpA

MKKNLRIVSVAAAALLAVAPVAASAVSTVSAADATTTTTATTTNKPTIDL

TGAGAVTNAADTVTVTPNFTLTAAVAKDKKVTASATLQGTITASLNGTSV

TANVIDAAKDVTLKSNTGYTTIYKYDSTTKTTENNLGKWNEATNDVYVKA

GNDYQVELTGVGFSFGSANANKELSLKLPSNVTVTGAAVNGNKLKLDQYG

NVTNLTFTVKDIKAYDATNTSAVQFYNTNSGLIESKAASYMALADNNGNL

NVNTLLDGLKKQYKAVQLQNGELKDVNVTTTAADLTAELEKAGIKVNAAG

DFEAPASFTATLTAKSTVNGKVATLPVTVTVPNGKVVPSQSKTIMHNAYF

YDKDAKRVGTDKVTRYNTVTVATSTTTINGKAYYEVVENGKATGKFINAD

NIDGTKRTLKHNAYVYKSSKKRANKVTLKKGTEVTTYGGTYTFKNGKQYY

KIGNNTEKTYVKASNF

>GRL1116_*slpA*

atgaagaaaaatttaagaattgttagcgttgctgctgctgctttattagc

tgttgctccagttgctgcttctgctgtatctactgtttcagctgctgacg

ctactacaactactactgctactacaaccaacaagccaactattgactta

actggtgcaggtgctgttactaacgcagctgatactgttactgtaactcc

taactttactttgactgctgctgttgctaaggataagaaagttactgctt

cagcaactttacaaggtactattactgcttcacttaacggtacttcagta

actgctaacgtaattgacgctgctaaggatgttactttaaagagtaacac

aggctacactactatttacaagtacgattctacaactaagaccactgaaa

acaaccttggtaagtggaacgaagcaactaacgatgtttacgtaaaagct

ggtaacgactaccaagttgaacttactggtgtaggcttcagctttggctc

agctaacgcaaacaaggaattatcacttaagttgccttcaaacgtaactg

ttacaggtgctgcagttaatggcaacaaattaaagttagaccaatacggt

aacgttactaacttaacctttactgtaaaggacattaaggcttacgatgc

tactaacactagtgctgttcaattctacaacactaactcaggcttaattg

aaagcaaggctgcatcatacatggctcttgctgacaacaacggtaacctt

aatgtaaacactcttcttgacggtttgaagaagcaatacaaagctgttca

acttcaaaatggtgaacttaaggacgttaatgtaaccactactgctgcag

accttactgctgaacttgaaaaggctggtattaaggtaaatgctgctggt

gactttgaagctcctgcatcattcactgcaactttaactgctaagtcaac

agttaacggtaaggttgctactttgcctgtaactgtaactgttccaaacg

gcaaggttgtaccaagccaaagcaagactattatgcacaacgcatacttc

tacgacaaggacgctaagcgtgttggtactgacaaggtaactcgttacaa

cactgtaactgttgcaacttcaaccactactatcaacggtaaggcttact

acgaagtagttgaaaacggcaaggcaactggcaagttcatcaacgcagac

aacattgacggtactaagcgtactttgaagcacaacgcatacgtttacaa

gtcttcaaagaagcgtgctaacaaggttaccttgaagaagggtactgaag

taactacttacggtggtacttacacattcaagaacggcaagcaatactac

aagatcggtaacaacactgaaaagacttacgtaaaggcttcaaactttta

a

>GRL1117_SlpC1

MKKNKLILASVAALMAVSPVLSFGSQVHTVQAADNSVRKTVMYNSIAYDK

DGNSTGQKYYTYGSISVDPTPVTINGNQYYKISGKNQYVRVTNIDGVRRR

VTHNAYIYRTSTQKTPYGMTASSKKWKLYKGEIVTTYGGYYTFKNGKHYF

KVGGPRKQYVRTANLGPVIGTNTSTSSNNSSNTPTNNTQSVGKYETTVTV

TTPYTYLFTEVPGKIQVQRTNKRVKKGDKFVVDRLEQGTRAGTGQDGDDD

NELAIYHIKGTDYWIYNNDVQAAKQLSVQSYNKTDKSLITMDQPVEVYNA

DGTSQNIRIKKNDSAWRVDSLSYIWVAKENKAELFYRLHLNGEYRSVYRL

TNNGDYVSDRVPIKNAYIKASEVKVDPNGLKLTPSDTAAEAEAAAKK

>GRL1117_*slpC1*

atgaagaagaataaacttatcttggcttcagttgcagcattaatggctgt

aagcccagtgctttcattcggctcacaagttcacacggttcaagctgcag

ataattctgtcagaaagacagttatgtataattcaattgcttatgataaa

gatggcaattcaacaggtcaaaagtattacacttacggatcaatcagtgt

tgatccaacccctgtaactattaacggtaaccaatattacaagatttcag

gtaaaaaccaatatgttagagtaactaatattgatggtgtaagacgtaga

gtaacccacaatgcttatatttatcgtacttctactcaaaaaacgcctta

cggtatgactgcaagcagtaagaaatggaagttatacaaaggcgaaatag

taactacttatggtggctattacacctttaaaaatggtaagcactacttc

aaggtaggcggaccaagaaagcaatatgttagaactgctaacttaggtcc

agttatcggaactaatacttcaacaagttctaataattcgtcaaacaccc

caactaataatacccaatcagttggtaaatatgaaaccactgtaacagta

actactccatacacttatctttttacagaagttccaggtaaaatccaagt

ccaacgtactaataaacgtgttaaaaaaggtgataaatttgtggtagacc

gtttagaacaagggacacgtgctggtactgggcaagacggtgatgacgat

aatgagctagcaatttatcatattaagggaacggattactggatttataa

taatgatgttcaagctgctaagcaattatcagttcagagctataacaaaa

cagacaaatcattaattactatggatcaaccagttgaagtctacaatgca

gatggtacttctcaaaatattagaattaagaagaacgattcggcatggag

agttgatagcttatcatacatttgggtagccaaggaaaataaggctgaac

tattctatcgtttacatttgaatggtgaatatagaagcgtttatcgctta

acaaacaatggcgactatgtttccgatcgtgttccaattaaaaatgcata

cataaaagcaagtgaagttaaagttgatccaaatggtttgaaattaacac

catcagacactgctgctgaagcagaggctgctgcgaaaaagtaa

>GRL1117_SlpC2

MKKNKLILASVAALMAVSPVLSFGSQTHTVQAADTTITKTIMHTSMAYDR

DGKSTGTKYYAYKTVDVMTKPVKINDNLYYKVNGLNHYLRATNIDGVTRK

VTHNTYVYKSSNGRTSYNGRWKLYKGETVTTYGGSYKFKNGKLYFRIGGP

SKQYIKSANLGPVIKINTSVNGSSSSSTSTNLEETTVTVTTPTRLITQTS

NGYKGTAHITPVGTKFTVDRLEFNELSKRSENDDHFYHIKGTNQWINASD

VKAAKSIPLHDYFFENFSYITFPKDTDVYNADGTIQDHNGQKISKQKGQL

KVDKLVYIWVPSENKAELFYHLVGTSFYASTTPTVHWSTINVGHNAYVKA

SDVKFIDGSVKLTPSNTAAEAEAAAKK

>GRL1117_*slpC2*

atgaagaaaaacaaacttatcttagcttcagttgcggctttgatggctgt

aagcccggtgctttcatttggctcacagactcacacagttcaagctgcag

acactactattactaaaacaatcatgcatacttcaatggcatatgacaga

gatggtaagagcacaggtaccaaatattatgcatacaagaccgttgatgt

aatgactaagcctgtaaaaataaatgacaatttgtattacaaagtaaatg

gactaaatcactatttaagagctaccaatattgatggtgtaacgcgtaaa

gtaacgcataacacttatgtttataagtcttccaatggtcgtacttcgta

taacggcagatggaagctttataaaggtgaaacagtcactacttatggtg

gttcttacaaattcaaaaatggcaagctctacttcagaattggtggtccc

agcaaacaatacattaaatcagcaaatttaggaccagttattaaaattaa

tacttcagtgaatggatctagttcatcatcaacatctactaatttggaag

aaacaacagtcactgtaaccactccaactcgtttaattacccaaactagt

aatggctataagggaacggctcatattactccggtaggaacaaaatttac

agttgatcgtttggaatttaatgaactttctaagagaagtgaaaacgacg

accatttttatcatatcaaaggtactaatcagtggattaatgcttctgat

gtgaaagctgctaagagtattccactacatgactatttctttgaaaactt

ctcatacattacattccctaaagatactgatgtgtataatgcagacggaa

ctatccaagatcataacggtcaaaaaataagtaaacaaaaaggccaattg

aaagtagacaagttagtatatatttgggtgccaagtgaaaataaggctga

attattctatcatttggtaggtacaagcttttatgcaagtacaacgccaa

ccgttcattggagcacaattaatgttgggcataatgcttatgtaaaagcc

agcgatgttaagtttattgacggaagcgtaaagctaactccatcaaacac

agccgctgaagcagaagctgctgcgaagaagtaa

>GRL1117_SlpB

MKKNRKMLGLAAAALLAVAPVVTSAVPVSADTPTVDTGLSGTVSSPAKSQ

VTGATPFFSYKNGAPIYSGGSTPNINAGSFTTIGQIVDAINNNIIFGEAG

STGTTRKEDISATEVIRQLKADSKDVIIHGKDANAKVSNLPANFTITLKH

TVNGQANTLNVLFYTTAQPTESVDKSAPVFYVTEGSSAAKQATSGAYYQV

AAGSNFNPLSFVNSNGETVSFSARQADGNNAGATVSVASNPVDTTNAGRF

YTVTLTATNTSNKTSRYSYTVLIVSNGLQKVYANGASSVATYSIYGNQVS

SNSTTFKDGQEVYVGNTTRTINNVSYSKVSTKSKADADQGNLWIQTSALT

QTNNTTTDSNTETHTVMIDSRAYDKNGNYLGHMYYAYDNIDIVPTVVTIN

GKTYYKVANKDEYVRVTNITGNTRTLTHNAYIYWSSYRRTPGTGKMYKGQ

TVTTYGPAMRFKNGKKYYRIEGCRNNNKRYIKAANLSAAQ

>GRL1117_*slpB*

atgaagaaaaatagaaaaatgttaggtttagctgctgccgctttgttagc

agttgcacctgttgtaactagtgctgtacctgtaagtgctgacacaccaa

cagttgatactggcttatcaggtactgtaagtagcccagcaaaatcacaa

gttaccggtgctaccccattcttctcatataagaatggtgccccaattta

ttctggcggtagcacaccaaacattaatgctggttcatttaccactattg

gtcaaatcgtagatgccatcaataacaacattatctttggtgaagctggc

tcaactggaacaactcgtaaagaagatatttcagctacagaagtaattag

acaattaaaggctgacagtaaggatgttataattcatggtaaggatgcaa

atgccaaagtttcaaacttacctgcaaactttaccattactttgaagcac

actgtaaatggtcaagctaacactttgaacgttttattctacactactgc

tcaaccaacagaatccgtagataagtctgccccagtattctacgtaactg

aaggttcatcagctgctaagcaagccacttcaggtgcatactaccaagta

gctgcaggctcaaacttcaacccattgagcttcgtaaacagtaatggtga

aactgtatcattctcagctcgtcaagctgatggtaataacgctggtgcaa

ctgtaagtgttgcttctaacccagtagatactaccaacgcaggtcgtttc

tacactgttactttgactgctactaacacttcaaacaagactagccgcta

ctcatacactgtattgattgtttcaaacggtttacaaaaagtttatgcaa

acggtgctagctcagtagcaacttacagcatttacggtaaccaagtttca

tcaaactcaactacttttaaggatggtcaagaagtttacgtaggtaacac

tacaagaactattaacaacgtatcatactcaaaggtatcaactaagtcta

aggcagatgctgatcaaggtaacctttggattcaaacttcagctttgact

caaactaacaacactactactgattcaaacactgaaacccatactgtaat

gattgactcacgtgcttacgacaagaacggtaactacttaggccacatgt

actacgcatatgacaacattgatatcgttccaactgttgtaaccatcaac

ggcaagacttactataaggttgctaacaaggatgaatatgttcgtgtaac

caacattactggtaacactcgtaccttgacccacaacgcttacatttact

ggtcatcatatcgtcgtaccccaggtactggtaagatgtacaaaggccaa

actgtaactacttacggtcctgcaatgagattcaagaacggtaagaagta

ctacagaattgaaggctgcagaaacaacaacaagcgttacatcaaggcag

ctaacttgagcgcagctcaataa

>GRL1117_SlpA1

MKKNLRIVSAAAAALLAVAPVAATAIATPATTVQAKVNADQVKAGAKLTL

TEDVKVVDVKTGHYHGTAAKKGETVTVKNVTKNKQYAILSNNKIVKVSDL

ANSNPAAPTENGSQSVTVKNNGTKIFNYTNNEFEDAKSTLPVGSTLTVSQ

KVYRQDGKAYYAILNSDGKATGQFVLVSDVTVAPDDKASENIVPSDNIFN

SIFENDPNVAVNISATASQDRNGAQINWYSTLNGQLIYTDAKGASHTALL

HGNENTVVYRAKTGERVTDHLEPGKQYVADFKKVTINLGNSAAGTSVKFI

LPRTARFAKGADKDYNNVKTVTVDSNGIADLGEVYAQFYAYNPTDLQEVH

FYSEKTGNQVTSGNINLHAVNGKLGLYSLFHAMNQEYVAAQLDGGKHATY

ASRTDMANRPQDMLPVAVQNDLRDQLKKQNITVDDNGYFTAPASFTLNMN

AKSIYNGATATLPVTINVDNAQASNTQETTKNVTIMHIATIYDKNGKATH

EPALRAYDTVSVVSDPVSLKDEKGNDAGKFYKLAGKDEYIKVGNVDGTSR

SLKHDSYVYKSTGKRNGKKVLKKGSSVTTYGKSFMIAGHQMYRIGKNQYV

KKANF

>GRL1117_*slpA1*

atgaagaaaaatttaagaattgttagcgctgctgctgctgctttattagc

tgttgctcctgttgctgctactgctattgctactccagcaactactgtac

aagctaaggttaatgcagatcaagttaaggccggcgcaaagcttacattg

actgaggatgtcaaagtagttgatgtaaagactggccactaccatggtac

tgctgctaagaagggtgaaacagtaactgttaagaacgttactaagaata

agcaatacgctattttatcaaacaataagattgttaaggtatctgacctt

gctaacagtaatccagctgctccaactgaaaatggttcacaatcagtaac

tgttaagaacaacggtactaagatcttcaactacactaacaacgagttcg

aagatgctaagagcactttgcctgttggctcaactcttactgtttcacaa

aaggtataccgtcaagatggcaaggcttactacgctattcttaactcaga

tggcaaggcaactggtcaatttgtattagtttcagatgtaaccgttgctc

cagatgacaaggcatcagaaaatattgtacctagtgacaatatcttcaac

tccatttttgaaaatgatccaaacgttgcagttaacatcagtgctactgc

ttcacaagacagaaatggtgctcaaatcaactggtacagcactcttaacg

gtcaacttatttacactgacgctaaaggtgcttctcacactgccttgtta

cacggcaacgaaaacactgttgtataccgtgctaagactggtgaaagagt

aactgatcacttagaacctggtaaacaatacgtagcagacttcaagaagg

ttactatcaaccttggtaactcagctgctggcacaagcgttaagtttatc

ttgccaagaactgcacgttttgcaaagggtgctgataaggactacaacaa

cgttaagactgtaactgttgattcaaatggtatcgcagatcttggtgaag

tttacgctcaattctacgcatacaacccaactgacttgcaagaagttcac

ttctactcagaaaagactggtaaccaagtaacttcaggtaacattaattt

acatgctgtaaacggtaagcttggcctttactcattgttccacgcaatga

accaagaatacgttgctgcacaacttgacggtggtaagcacgcaacttac

gcatcacgtacagacatggcaaacagacctcaagatatgttaccagttgc

tgtacaaaacgacttaagagatcaattgaagaagcaaaacatcactgttg

atgacaatggttacttcactgctccagcatcattcactcttaacatgaac

gctaagtcaatttacaacggtgctactgcaactttgcctgtaactatcaa

cgttgacaatgctcaagcttcaaacactcaagaaactactaagaacgtaa

ctatcatgcacattgcaaccatctacgacaagaatggtaaagcaacccat

gaaccagcattacgtgcttacgacactgtatcagtagtttcagacccagt

ttcattgaaggatgaaaagggtaacgatgcaggtaagttctacaagcttg

ctggcaaggatgaatacatcaaggttggtaacgttgacggtacttcacgt

tcattgaagcacgactcatacgtttacaagtcaactggtaagagaaacgg

caagaaggttcttaagaagggctcatcagtaactacttacggtaagtcat

tcatgattgctggtcaccaaatgtacagaatcggcaagaaccaatacgtt

aagaaggcaaacttctaa

>GRL1117_SlpA2

MKKNLRIVSAAAAALLAVAPVAATVVPTATANAAVAIKGNTLATGDINLS

LKKDQKLSDANTLSALHNEAVARITNQVAKSIHMDPSQVTIKADWSGLVN

NAEKVTYTVSVPHTNWAPGKVTSDGTHQIGNYRWNKQGQIVDASEDNAAD

AKAAEFTATVNYKLAKESDASSENIVTDGSGIVNSVFENDPNVSVNINAT

AETAGNRVVNWASVLNGQLIYTDAKGTSHTALLHGDNNTTVYKVSDFKKG

KVVAGTTPQKSMTAGQQYVADFNKVTINLGSSAAGSKVKFVLPRTAWFAK

HDKNYNNVQTVTVDSNGVADLGTVYAQFYAYDPTDLQEIHFYSEKTGNQV

TSGNINLHAVNGKLGLYSLFHAMNYEYVAAQLNGGKGADYAKQPGYYSDN

MLPVAVQNDLRDQLKKQNITVDDNGYFTAPASFTVNMNAKSIYNGATATL

PVTVTVDNVTPTAANETTKNVTIMHIATIYDKNGKATHEPALRAYNSVSV

VSDPVSLKDEKGNDAGKFYKLAGKDEYIKVGNVDGTSRSLKHNSYVYKST

GKRNGKKVLKKGSSVTTYGKSFMIAGHQMYRIGKNQYVKKANF

>GRL1117_*slpA2*

atgaagaaaaatttaagaattgttagcgctgctgctgctgctttattagc

tgttgctcctgttgctgctactgtagttccaaccgctactgctaacgctg

ctgtagcaattaagggtaacactcttgctactggtgatatcaacttaagc

ttaaagaaggatcaaaagttaagtgatgctaatacactttcagctcttca

taacgaagctgtagctagaattactaaccaagttgcaaagtcaattcaca

tggatccttcacaagttaccattaaggctgactggagcggcttagttaac

aacgcagaaaaagtaacttatactgtttctgttccacatactaattgggc

tccgggtaaagttactagtgatggtacacaccaaattggtaactacagat

ggaacaagcaaggtcaaattgtagatgcttctgaagataacgctgctgat

gctaaagctgctgaatttactgcaactgtaaactacaagctagctaagga

atctgacgcttcatcagaaaacattgttactgatggtagtggcattgtta

actctgtctttgaaaacgatccaaacgtttctgttaacatcaacgctact

gctgaaacagctggtaatcgagttgtaaactgggctagtgttcttaacgg

tcaacttatttacactgatgctaagggtacttcacacactgctttattac

acggtgataacaacactactgtatacaaggtaagtgatttcaagaagggt

aaagtagttgctggtactacaccacaaaagagtatgacagcaggtcaaca

atacgtagcagacttcaacaaggttactattaaccttggtagctcagctg

ctggctcaaaggttaagtttgtattgcctagaactgcatggtttgctaag

catgacaagaactacaacaacgttcaaactgtaactgttgattcaaacgg

tgttgcagaccttggtactgtttatgctcaattctacgcatacgacccaa

ctgatttacaagaaatccacttctactcagaaaagactggtaatcaagta

acttcaggcaacatcaatttacatgctgtaaacggtaagcttggtcttta

ctcattgttccacgcaatgaactatgaatacgttgctgcacaacttaacg

gtggtaagggcgcagattacgcaaaacaaccaggttactactcagataac

atgttaccagttgctgtacaaaatgaccttagagatcaattgaagaagca

aaacatcactgttgatgacaatggttacttcactgctccagcatcattca

ctgtaaacatgaacgctaagtcaatttacaacggtgctactgcaactttg

cctgtaactgtaactgttgacaacgtaactccaactgctgctaacgaaac

tactaagaacgtaactatcatgcacattgcaaccatctacgacaagaatg

gtaaggcaacccatgaaccagcattacgtgcttacaactcagtatcagtt

gtttcagacccagtttcattgaaggatgaaaagggtaacgatgcaggtaa

gttctacaagcttgctggcaaggatgaatacatcaaggttggtaacgttg

acggtacttcacgttcattgaagcacaactcatacgtttacaagtcaact

ggtaagagaaacggcaagaaggttcttaagaagggctcatcagtaactac

ttacggtaagtcattcatgattgctggtcaccaaatgtacagaatcggca

agaaccaatacgttaagaaggcaaacttctaa

>GRL1118_SlpC1

MKKNKLILASVAALMAVSPVLSFGSQVHTVQAADNSVRKTVMYNSIAYDK

DGNSTGQKYYTYGSISVDPTPVTINGNQYYKISGKNQYVRVTNIDGVRRR

VTHNAYIYRTSTQKTPYGMTASSKKWKLYKGEIVTTYGGYYTFKNGKHYF

KVGGPRKQYVRTANLGPVIGTNTSTSSNNSSNTPTNNTQSVGKYETTVTV

TTPYTYLFTEVPGKIQVQRTNKRVKKGDKFVVDRLEQGTRAGTGQDGDDD

NELAIYHIKGTDYWIYNNDVQAAKQLSVQSYNKTDKSLITMDQPVEVYNA

DGTSQNIRIKKNDSAWRVDSLSYIWVAKENKAELFYRLHLNGEYRSVYRL

TNNGDYVSDRVPIKNAYIKASEVKVDPNGLKLTPSNTAAEAEAAAKK

>GRL1118_*slpC1*

atgaagaagaataaacttatcttggcttcagttgcagcattaatggctgt

aagcccagtgctttcattcggctcacaagttcacacggttcaagctgcag

ataattctgtcagaaagacagttatgtataattcaattgcttatgataaa

gatggcaattcaacaggtcaaaagtattacacttacggatcaatcagtgt

tgatccaacccctgtaactattaacggtaaccaatattacaagatttcag

gtaaaaaccaatatgttagagtaactaatattgatggtgtaagacgtaga

gtaacccacaatgcttatatttatcgtacttctactcaaaaaacgcctta

cggtatgactgcaagcagtaagaaatggaagttatacaaaggcgaaatag

taactacttatggtggctattacacctttaaaaatggtaagcactacttc

aaggtaggcggaccaagaaagcaatatgttagaactgctaacttaggtcc

agttatcggaactaatacttcaacaagttctaataattcgtcaaacaccc

caactaataatacccaatcagttggtaaatatgaaaccactgtaacagta

actactccatacacttatctttttacagaagttccaggtaaaatccaagt

ccaacgtactaataaacgtgttaaaaaaggtgataaatttgtggtagacc

gtttagaacaagggacacgtgctggtactgggcaagacggtgatgacgat

aatgagctagcaatttatcatattaagggaacggattactggatttataa

taatgatgttcaagctgctaagcaattatcagttcagagctataacaaaa

cagacaaatcattaattactatggatcaaccagttgaagtctacaatgca

gatggtacttctcaaaatattagaattaagaagaacgattcggcatggag

agttgatagcttatcatacatttgggtagccaaggaaaataaggctgaac

tattctatcgtttacatttgaatggtgaatatagaagcgtttatcgctta

acaaacaatggcgactatgtttccgatcgtgttccaattaaaaatgcata

cataaaagcaagtgaagttaaagttgatccaaatggtttgaaattaacac

catcaaacactgctgctgaagcagaggctgctgcgaaaaagtaa

>GRL1118_SlpC2

MKKNKLILASVAALMAVSPVLSFGSQTHTVQAADTTITKTIMHTSMAYDR

DGKSTGTKYYAYKTVDVMTKPVKINGNLYYKVNGLNHYLRATNIDGVTRK

ITHNTYIYKSSNGRTSFNGRWKLYKGETVTTYGGSYKFKNGKHYFRIGGP

SKQYIKSANLGPVIKTNTSVNGSGSSSASTNSEETTVTVTTPTRLITQTS

NGYKGTAHITPVGTKFTVDRLEFNELSKRSENDDHFYHIKGTDQWINASD

VKAAKSIPLHDYFFENFSYITFPKDTDVYNADGTIQDHNGQKISKQKGQL

KVDKLVYIWVPSENKAELFYHLVGTSFYASTTPTVHWSTINVGHNAYVKA

SDVKFIDGSVKLTPSNTAAEAEAAAKK

>GRL1118_*slpC2*

atgaagaaaaacaaacttatcttagcttcagttgcggctttgatggctgt

aagcccggtgctttcatttggctcacagactcacacagttcaagctgcag

acactactattactaaaacaatcatgcatacttcaatggcatatgacaga

gatggtaagagcacaggtaccaaatattatgcatacaagaccgttgatgt

gatgactaagcctgtaaaaataaatggcaatttgtattacaaagtaaatg

gattaaatcactatttaagagctaccaatattgatggtgtaacgcgtaaa

ataacgcataatacctatatttataaatcttctaatggtcgaacttcatt

taatggtagatggaagctttataaaggtgaaacagtcactacttatggtg

gttcttacaaattcaaaaatggtaagcactacttcagaattggtggtcca

agcaaacaatacattaaatcagccaatttaggaccagttattaaaactaa

tacttcagtgaatggatctggttcatcatcagcatctactaattcggaag

aaacaacagtcactgtaaccactccgactcgtttaattacccaaaccagt

aatggctataaaggaacggctcatattactcctgtaggaacaaaatttac

agttgatcgtttggaatttaatgaactttctaagagaagtgaaaacgacg

accatttttatcacatcaaaggtactgatcagtggatcaatgcttctgat

gtgaaagctgctaaaagtattccactacatgactatttctttgaaaactt

ctcatacattacattccctaaggacactgatgtgtataatgcagacggaa

ctatccaagatcataacggccaaaaaataagtaaacaaaaaggtcaatta

aaagtagacaagttagtatatatttgggtaccaagtgaaaataaggctga

attattctatcacttggtaggcacaagtttttatgcaagtacaacgccaa

ccgttcattggagcacaattaatgttggtcataatgcttatgtaaaagct

agcgatgttaaatttattgatggaagcgtaaaattaactccatcaaacac

agccgctgaagcagaagctgctgcaaagaagtaa

>GRL1118_SlpB

MKKNLRIISVAAAALLAVAPVATSVVPTVGANVVQAADNTITGSVVNNSNA

ATGNTAANQGTTTNIANAPTNRPFFARNYEAIGEATTENPNANVVRQTVSI

KVGETADDIAKDLKNLKIVFHPSVSDTKTTAFAPSADEVVKLLNNVTFETP

KSGANKGKQVVKTLPNGDFNLTITGQANNQTAAIQIPFVVSSSSSATDTTG

NPVISYTVAGQAGKNPVFQVAANSQFNPLDFTNAAGDEVKFSAVQASGSSI

QASLTATSNPVDTSQQGRFYNVTLTATNLNGRTSTFTYTVLITSSQKQTLY

GNGTINTYNIYGNNALNGSTTFKSGDTVYVADATKTINGVSYSQVSTKSKA

DAATSNIWVKTADLTKPATPSDTNVKTYPVMIDSRAYDKNGNYLGHMYYAY

DSIDIVPTVVNIKGKTYYKVANKDEYVRVTNITGNKRTLTHNAYIYWSSYR

RTPGTGKMYKGQTVTTYGPAMRFKNGKKYYRIEGCRNNNKRYIKAANLSAA

Q

>GRL1118_*slpB*

atgaagaaaaatttaagaattattagtgttgcagctgctgctttattagc

agttgctccggtcgcaacttcagttgtacctactgttggtgctaatgttg

ttcaagctgctgataacactattactggtagtgtcgttaataacagcaat

gctgcaactggtaatactgctgctaaccaaggtactactactaatattgc

caatgcaccaactaatcgtccattctttgctagaaactatgaagcaattg

gagaagcaacaactgaaaatccaaatgctaacgttgttcgtcaaactgta

agtattaaagtgggcgaaactgctgatgatattgctaaagatcttaagaa

tttgaagattgttttccacccctcagtaagtgatactaagaccaccgctt

ttgcaccaagcgcagatgaagtagtaaagttattgaacaatgtaactttt

gagactccaaagagtggagctaacaagggtaagcaggtagttaaaacttt

accaaatggtgactttaacttaactattactggtcaagctaataaccaaa

ctgctgccattcaaattccatttgtagtttcatcatcttctagtgcaact

gataccactggtaaccctgtaatttcttacactgttgctggtcaagctgg

taagaacccagtattccaagttgctgctaattcacaatttaacccattag

actttactaatgctgctggtgatgaagttaaatttagcgcagttcaagct

tcaggtagctcaatccaagctagcttaactgcaacttcaaaccctgtaga

tacttcacaacaaggtcgtttctataacgtaactttgactgcaactaact

tgaacggtagaacaagcacctttacttacactgttttaattacttcaagt

caaaagcaaactttgtacggtaatggtactattaacacctacaacatcta

tggtaacaacgctttaaacggttcaactactttcaagagtggtgacactg

tttatgtagctgacgctactaagactattaacggtgtatcatactcacaa

gtttcaactaagtcaaaggctgatgctgcaacaagcaacatctgggtaaa

aactgctgatttgactaaaccagctaccccaagtgatactaacgttaaga

cttacccagtaatgattgactcacgtgcttacgacaagaacggtaactac

ttaggccacatgtactacgcatatgacagcattgatatcgttccaactgt

tgtaaacatcaaaggcaagacttactacaaggttgctaacaaggatgaat

acgttcgtgtaaccaacattactggtaacaagcgtaccttgacccacaat

gcttacatttactggtcatcataccgtcgtaccccaggtactggtaagat

gtacaaaggccaaactgtaactacttacggtcctgcaatgagattcaaga

acggtaagaagtactacagaattgaaggctgcagaaacaacaacaagcgt

tacatcaaggcagctaacttgagcgcagctcaataa

>GRL1118_SlpA

MKKNLRIVSVAAAALLAVAPVAASGVVAPTAVVSAADATNSNADYSHINL

GGSDVAKYVANVNPSFTLNAALRKNNANTDPNAQAVAAGSLTGSVTANVG

GVTATANLVNGDHGVADVKVTAVQGGTVIYDGTDAAHVVSNFNAVVAGQK

YSIVVNKVGFNFGANNAGKEVTLALPKNVDVVFSAAESGWTVGNDGKTLK

GKLDNNGTVNNIQLTETVTAFDASNTNAVVFYNIATGQQVNSGNTMVLAD

YNGQLNVNSILQAIKSNFTAFQRVTTPNNSEQNGSENQNKFDNPNIVTTV

DEIKDQLEKAGIKVNAADNFNAPHSFTVTVKAVSDINGKDAKLPVTFTVA

NVADPVVPSQTKTIMHNAYYYDKDAKRVGTDKVTRYNTVAVATSTTKIGD

KTYYEVVENGKLSGKFINADNIDGTKRTLKHNAYVYASSKKRANKVVLKK

GTEVTTYGGSYTFKNGKQYYKIGNNTDKTYVKASNF

>GRL1118_*slpA*

atgaagaaaaatttaagaatcgttagcgttgctgctgctgctttacttgc

cgttgctccagttgctgcttctggcgttgttgcacctactgcagttgttt

ctgctgctgatgctactaacagtaatgcagattattcacacattaattta

ggtggatcagacgttgctaagtatgttgcaaacgttaacccatcatttac

tttaaacgctgctcttcgtaagaacaacgctaacactgatccaaatgctc

aagctgttgctgcaggtagcttgactggtagtgttactgctaacgttggt

ggcgtaactgctactgctaacttagttaacggtgatcacggtgtagctga

cgttaaggttactgctgtacaaggcggtactgtaatttatgatggtactg

atgctgctcacgttgtatcaaacttcaacgctgtagttgctggtcaaaag

tacagcatcgttgttaacaaggttggcttcaactttggtgctaacaatgc

aggtaaggaagtaacacttgctttaccaaagaatgttgacgttgtgttct

cagcagctgaatcaggctggactgtaggcaacgacggcaagaccttaaag

ggtaagttagacaacaatggtactgttaacaacattcaattaacagaaac

tgttactgcttttgatgcttcaaacactaatgctgtagtattctacaaca

tcgctactggtcaacaagtaaactcaggtaacactatggtacttgcagat

tacaatggccaacttaatgtaaactcaattttacaagctattaagagtaa

ctttactgcattccaacgtgtcactactccaaacaatagtgaacaaaatg

gttcagaaaaccaaaacaagtttgacaaccctaatattgtaactactgtt

gatgaaattaaggatcaattggaaaaggctggcatcaaggttaacgctgc

agataactttaatgctcctcactcattcactgtaactgttaaggctgttt

cagacattaacggtaaggatgcaaaacttcctgtaacctttactgtagct

aacgttgcagatcctgttgttccaagtcaaactaagactattatgcacaa

cgcatactactacgacaaggatgctaagcgtgttggtactgacaaggtaa

ctcgttacaacactgtagctgttgcaacttcaactactaagatcggtgac

aagacttactacgaagtagttgaaaacggcaagctttcaggcaagttcat

caacgcagacaacatcgatggtactaagcgtactttgaagcacaacgctt

acgtttacgcatcatcaaagaagcgtgctaacaaggttgttcttaagaag

ggtactgaagtaactacttacggtggttcatacaccttcaagaacggtaa

gcaatactacaagatcggtaacaacactgacaagacttacgttaaggctt

caaacttttaa
