# Supplementary figures and images for: Functional characterization of probiotic surface layer protein-carrying Lactobacillus amylovorus strains
Source: BMC Microbiol. 2014 Jul 28;14:199. doi: 10.1186/1471-2180-14-199 (PMC4236617; doi:10.1186/1471-2180-14-199)

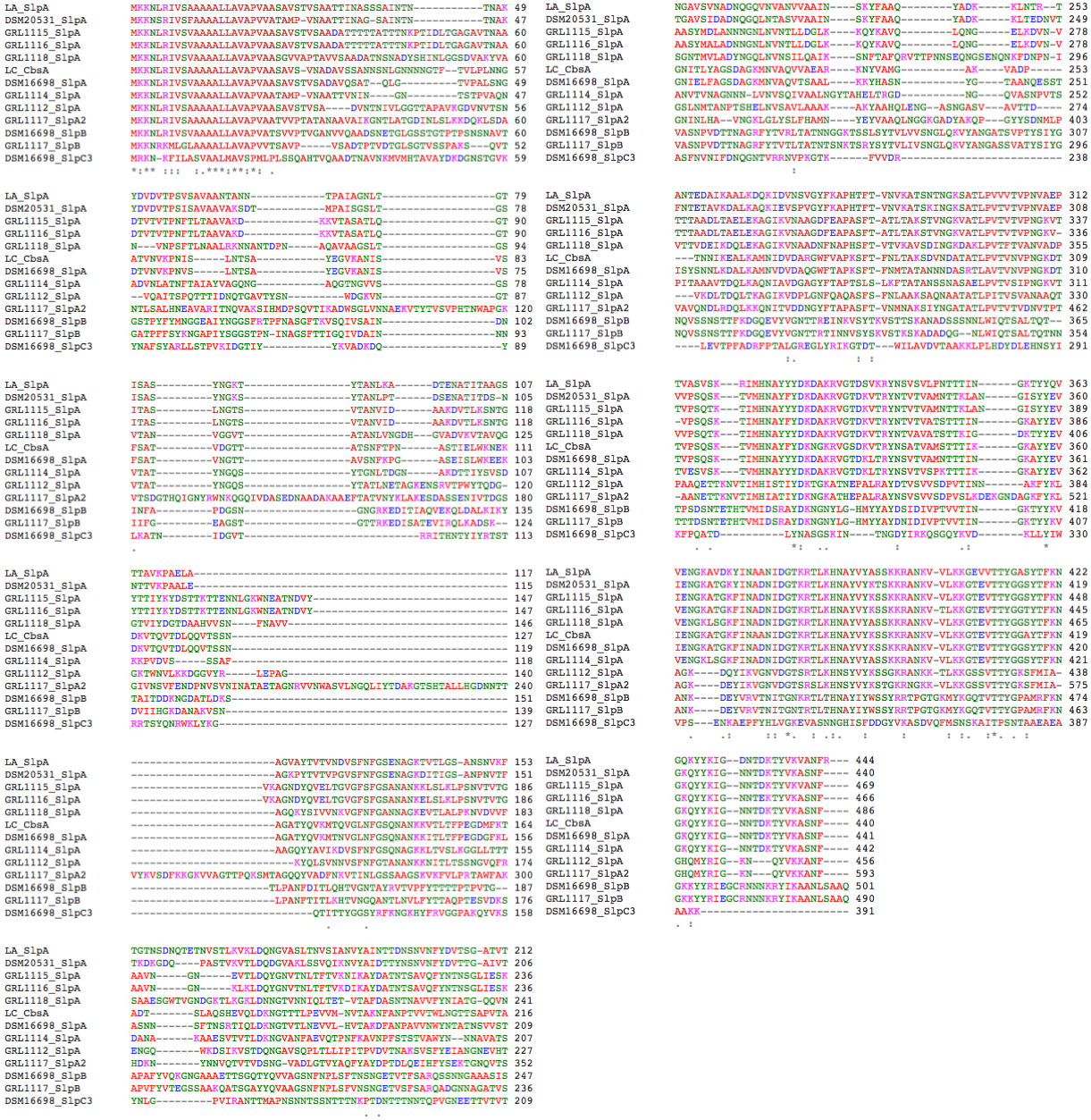

Supplement: Additional file 2 — Amino acid sequence alignment of L. amylovorus, L. acidophilus and L. crispatus S-layer proteins. A .tiff-image showing the alignment of the amino acid sequences of the surface-located Slp:s of the L. amylovorus strains studied, SlpA of L. acidophilus NCFM (LA, GenBank AAV42070) and CbsA of L. crispatus JCM 5810 (LC, GenBank AF001313), aligned by ClustalW available at http://www.ebi.ac.uk/Tools/msa/clustalw2/. [file 1471-2180-14-199-S2.tiff]
